# Extracellular Peptide-Ligand Dimerization Actuator Receptor Design for Reversible and Spatially Dosed 3D Cell-Material Communication

**DOI:** 10.1021/acssynbio.4c00482

**Published:** 2024-12-20

**Authors:** Matthias Recktenwald, Ritankar Bhattacharya, Mohammed Mehdi Benmassaoud, James MacAulay, Varun M. Chauhan, Leah Davis, Evan Hutt, Peter A. Galie, Mary M. Staehle, Nichole M. Daringer, Robert J. Pantazes, Sebastián L. Vega

**Affiliations:** † Department of Biomedical Engineering, 3536 Rowan University, 201 Mullica Hill Rd, Glassboro, New Jersey 08028, United States; ‡ Department of Orthopaedic Surgery, Cooper Medical School of Rowan University, Camden, New Jersey 08103, United States; § Department of Chemical Engineering, 1383 Auburn University, Auburn, Alabama 36849, United States

**Keywords:** synthetic biology, transmembrane receptors, biomaterials, peptides, computational protein design

## Abstract

Transmembrane receptors that endow mammalian cells with the ability to sense and respond to biomaterial-bound ligands will prove instrumental in bridging the fields of synthetic biology and biomaterials. Materials formed with thiol-norbornene chemistry are amenable to thiol-peptide patterning, and this study reports the rational design of synthetic receptors that reversibly activate cellular responses based on peptide-ligand recognition. This transmembrane receptor platform, termed Extracellular Peptide-ligand Dimerization Actuator (EPDA), consists of stimulatory or inhibitory receptor pairs that come together upon extracellular peptide dimer binding with corresponding monobody receptors. Intracellularly, *Stimulatory EPDAs* phosphorylate a substrate that merges two protein halves, whereas *Inhibitory EPDAs* revert split proteins back to their unmerged, inactive state via substrate dephosphorylation. To identify ligand-receptor pairs, over 2000 candidate monobodies were built *in silico* using PETEI, a novel computational algorithm we developed. The top 30 monobodies based on predicted peptide binding affinity were tested experimentally, and monobodies that induced the highest change in protein merging (green fluorescent protein, GFP) were incorporated in the final EPDA receptor design. In soluble form, stimulatory peptides induce intracellular GFP merging in a time- and concentration-dependent manner, and varying levels of green fluorescence were observed based on stimulatory and inhibitory peptide-ligand dosing. EPDA-programmed cells encapsulated in thiol-norbornene hydrogels patterned with stimulatory and inhibitory domains exhibited 3D activation or deactivation based on their location within peptide-patterned hydrogels. EPDA receptors can recognize a myriad of peptide-ligands bound to 3D materials, can reversibly induce cellular responses beyond fluorescence, and are widely applicable in biological research and regenerative medicine.

## Graphical abstract

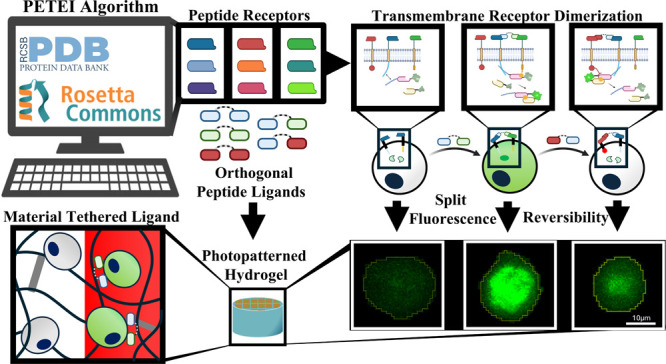

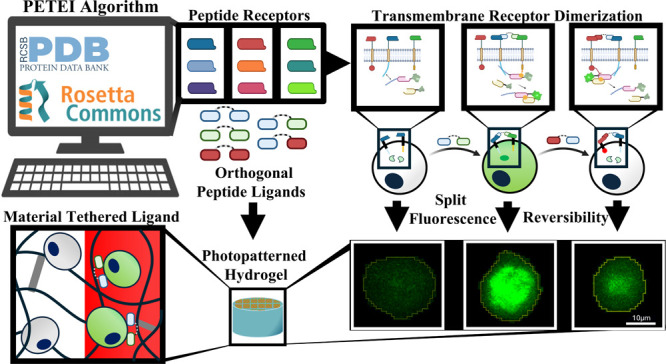

## 1 Introduction

Transmembrane receptors allow mammalian cells to actuate an external input by transmitting the signal across the membrane and into the cell.^1^ Biomaterials research utilizes biologically relevant chemical stimuli and physical cues to investigate cellular responses and to guide the development of therapeutic interventions.^2,3^ Biomaterials research has made tremendous advances in functionalizing biocompatible materials with biologically relevant peptide ligands that initiate native pathways.^4,5^ However, to-date, there has been no progress in the development of peptide material-receptor interactions which act independently of native pathways.^6,7^ Conversely, advances in synthetic biology have shown the clinical potential of programming mammalian cells with engineered transmembrane receptors which activate non-native pathways.^8^ These receptors have been designed to respond to soluble and cell-laden ligands; however, limited progress has been made in the design of synthetic receptors capable of responding to material-laden ligands.^9,10^ Synthetic transmembrane receptor systems which can respond to unique signals in the extracellular environment can be used to produce living materials with synthetic homeostatic biological feedback.^7^ These living materials will allow for better models for development and disease progression. Therapeutic peptides,^11^ regenerative biomaterials,^12^ and transmembrane receptor-based cell therapies^13^ have excelled over the past decade independently, and the development of material-laden peptide-ligands which activate synthetic pathways in engineered cells would allow for significant progress in the field of regenerative medicine.

Hydrogels are soft 3D biomaterials that are commonly used for studying cell-material interactions due to their biocompatibility and tunability over biophysical and biochemical properties.^14^ The formation of hydrogels consists of a transition from liquid to a solid polymeric network resulting from physical or chemical cross-linking mechanisms.^15^ To establish the role of specific material properties on cell behavior, it is important to use hydrogel synthesis schemes that permit the systematic variation of one material parameter at a time. Photopolymerization reactions that use click chemistry including Diels–Alder,^16^ azide–alkyne,^17^ and thiol-norbornene^18^ can be used to form hydrogels amenable to photopatterning reactions that can couple bioactive molecules to the hydrogel network with spatial and temporal control.^19–21^ Specifically, norbornene-modified hyaluronic acid (HyaNor) macromers can react with dithiolated cross-linkers to form HyaNor hydrogels that can undergo additional reactions between pendant norbornenes in the hydrogel network and monothiolated molecules.^18^ Peptides are short amino acid (AA) sequences usually designed to mimic natural proteins, and by including the thiol-containing AA cysteine, peptides can be covalently bound to HyaNor hydrogels with spatial control.^18,22^ While peptide-laden 3D HyaNor hydrogels have been used to study matrix mechanosensing,^23^ prevent bacterial growth,^24,25^ and to spatially regulate stem cell differentiation,^26^ these cell-material interactions activate native pathways, making it challenging to precisely control cellular responses. Peptide-ligand induced activation of synthetic receptor pathways would overcome this limitation.

Over the past three decades, synthetic biologists have taken a page from nature’s playbook and developed synthetic transmembrane receptors that sense extracellular inputs and convert these signals into user-defined cellular responses.^27,28^ Perhaps one of the most exciting examples of this is the development of CAR (chimeric antigen receptor) T-cell therapy which endows immune cells with the ability to identify and fight cancer.^29,30^ Since the development of CARs, a myriad of synthetic transmembrane receptors have been reported that detect surface-bound and soluble extracellular inputs. For example, in programmed T-cells, CARs transmit an intracellular signal using the endogenous T-cell activation pathway, whereas synNotch (synthetic Notch) receptor activation releases a transcription factor through γ-secretase mediated cleavage at the cell membrane. Both CARs and synNotch receptors rely on mechanosensing to actuate an external signal into an intracellular response. These receptors are used to target cell bound antigens which can generate the force required to initiate these responses. In contrast, receptors which rely on the dimerization of two receptors to relay an external signal such as MESA (Modular Extracellular Sensor Architecture)^31–33^ and GEMS (Generalized Extracellular Molecule Sensor)^33^ do not require these mechanical cues and are better suited for responding to soluble signals such as cytokines. While cell surface and soluble ligands have been explored, there is limited work looking at the targeting of material bound ligands. To our knowledge, to-date, fluorescent proteins are the only ligands used within biomaterials for activating engineered transmembrane receptors.^34^ This is an excellent use case for systems like synNotch to be adapted into biomaterials and developmental biology research.^35,36^ However, these relatively large fluorescent molecule ligands have the potential to be immunogenic,^37^ thereby limiting the capabilities of using biomaterials to manipulate cells programmed with synthetic receptors. Recent advances in peptide synthesis technology have allowed for cost-effective peptide production at scale,^38,39^ and can be easily incorporated to soft and rigid materials with spatiotemporal control via thiol-norbornene click chemistry reactions.^18,26,40^

With the goal of creating a transmembrane receptor that senses and responds to material-bound molecules, epitope tag peptides were selected as ligands. Epitope tags are short, orthogonal AA sequences used to isolate proteins for manufacturing, or to label proteins for localization studies.^41^ The most common and widely recognized of these are the FLAG, MYC, and HA epitope tags.^42^ The established use of these affinity tags in recombinant protein manufacturing makes them ideal candidates for ligands because they are not expected to induce a stress response in mammalian cells. Peptides which include these orthogonal tags can be designed to include a cysteine AA which allows for their use in thiol-norbornene click chemistry reactions. Since endogenous receptors are unable to sense epitope tag peptides, novel receptors need to be developed. Monobodies are based on the 10th type III fibronectin domain^43^ and share significant structural similarities with the binding domains of antibodies. However, monobodies are particularly well suited for cellular binding applications due to their lack of disulfide bonds,^44^ but we did not have access to monobodies for the epitope tags of interest. Therefore, we developed the PETEI (Proteins Designed with Targeted Exceptional Interactions) algorithm, which was inspired in part by the experimentally validated OptCDR^45,46^ and OptMAVEn^47,48^ algorithms for designing antibodies, to design monobodies that bind to HA, FLAG, and MYC epitope tag peptide sequences. To evaluate the utility of these peptide ligands for use in transmembrane receptor systems, we adapted a recently developed push–pull post translational circuit,^49^ and created a novel peptide-sensing transmembrane receptor termed EPDA (Extracellular Peptide-ligand Dimerization Actuator). Upon extracellular activation, intracellularly we adapted a reconstitution scheme that uses phosphorylation of a substrate to bring two fluorescent protein halves together.^50^ Substrate phosphorylation can be reversed by introducing a phosphatase that removes the phosphoryl group from the substrate, resulting in the dissociation of fluorescent protein halves, thereby deactivating the system.

Once candidate monobodies, based on binding affinity to either HA, FLAG, or MYC epitopes were determined *in silico*, these monobodies were incorporated into EPDA receptor designs which were tested experimentally. Stimulatory EPDA receptors consist of an HA-specific monobody and an intracellular zipper domain, and a FLAG-specific monobody which includes an intracellular kinase. For the Inhibitory EPDA receptor design, the FLAG-specific monobody and intracellular kinase domain were substituted with a MYC-specific monobody and intracellular phosphatase domain, which removes the phosphoryl group from activated, phosphoryl-bound substrates. To evaluate EPDA receptor communication within 3D materials, EPDA-programmed HEK293 cells were encapsulated in HyaNor hydrogels patterned with stimulatory and inhibitory peptide ligands, and we found that cells exhibited 3D activation or deactivation based on their location within peptide-patterned HyaNor hydrogels.

## 2 Results and Discussion

### 2.1 Intracellular Post-translational Phosphorylation-Based Circuit Design

To report extracellular dimerization events being transmitted through the membrane, we repurposed a synthetic phosphorylation-based circuit to induce split fluorescent protein reconstitution.^39^ This circuit utilizes various components found in nature to activate a synthetic phosphorylation event and phosphorylation-dependent recruitment. These components include a Zap-70 Src Homology 2 (SH2)^51^ domain which selectively binds to the phosphorylated tyrosine AA,^52^ a CD3ζ^53^ substrate domain containing tyrosine groups which are phosphorylated in native T-cell pathways, and an ABL-I kinase^54^ activation domain which selectively phosphorylates tyrosine (Figure 1 **A-C**). A basic region leucine zipper (LZEE)^55^ was also added to three repeats of the CD3ζ tyrosine domain. This allows for the kinase to act when it is brought into proximity with the substrate using the corresponding acidic region leucine zipper (LZRR), adding selectivity to the phosphorylation process (Figure 1 **Di**). As a positive control, the kinase and LZRR were attached together to determine the fluorescence expected from a constitutively dimerized zipper and kinase (CDZK) (Figure 1C). As a means of easily detecting this phosphorylation (Figure 1 **Dii**) and subsequent SH2 dimerization (Figure 1 **Diii**) event, split mNeonGreen^56^ green fluorescent protein (GFP) halves are attached to the tyrosine and zipper domains (Phosphorylation Recipient, PR) (Figure 1A) and SH2 domain (Phosphorylation Detector, PD) (Figure 1B). The PD will bind to the PR once it is phosphorylated and the split GFP molecule will be reconstituted (Figure 1 **Div**) which can be measured using flow cytometry or confocal microscopy. These GFP halves are shown to have weak but non-negligible background fluorescence when not reconstituted^56^ but could be replaced with other reporters such as split luciferase to reduce noise and allow for *in vivo* diagnostics. Glycine/serine linkers of 10AA, 10AA, 7AA, and 20AA on the PR, and a 10AA linker on the PD were placed between components to allow for phosphorylation, phosphorylation induced binding, and split protein reconstitution. These specific linker lengths were optimized in previous studies (unpublished). A constitutively dimerized zipper and PTP 1B phosphatase^57,58^ domain (ZPC) (Figure 1E**)** is used in receptor experiments to remove phosphoryl groups from the tyrosine residues on the PR (Figure 1 **Fi**). This results in the dissociation of the SH2 substrate complex and reduced fluorescent protein emission (Figure 1 **Fii**) ensuring that the fluorescence accounts only for continued proximity over the course of the ligand administration. These internal components and phosphorylation circuit will be used to report extracellular ligand-induced receptor dimerization throughout this manuscript. To develop materials capable of inducing this circuit, peptides were selected for these ligands because they can be easily conjugated to many biomaterials through click chemistry reactions as discussed in the upcoming section.

**Figure 1:**
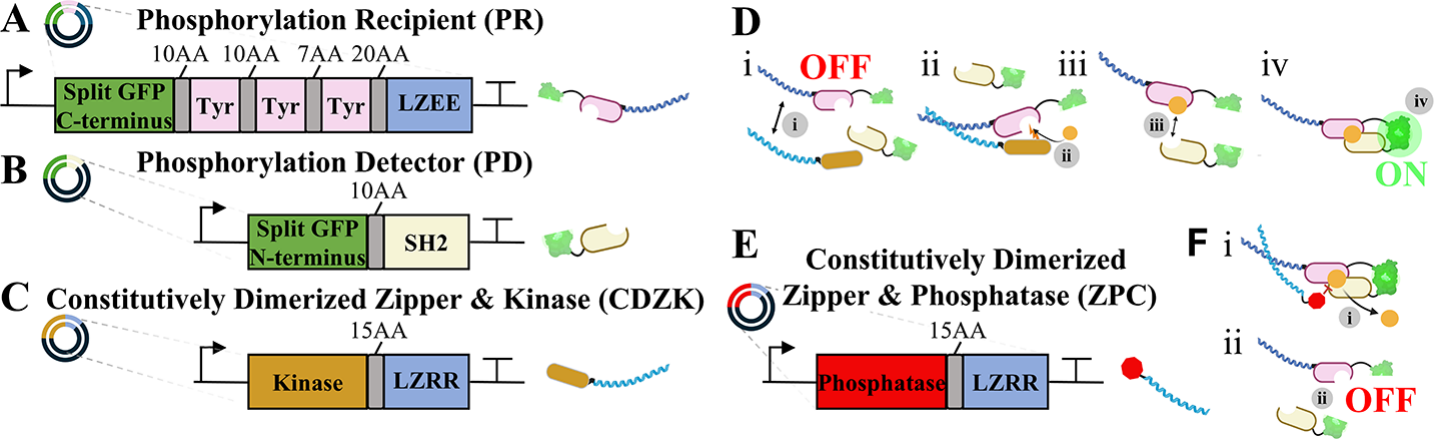
**Intracellular phosphorylation-based circuit components and activation/deactivation workflows.** (A) The phosphorylation recipient (PR) is comprised of three CD3ζ subunits containing tyrosine-based activation motifs (Tyr), a leucine zipper, and the C-terminus of a split mNeonGreen green fluorescent protein (GFP). (B) The phosphorylation detector (PD) is comprised of an Src Homology 2 (SH2) domain and the N-terminus of a split GFP molecule. (C) The constitutively dimerized zipper and kinase (CDZK) component consists of an ABL-I kinase domain and an acidic region leucine zipper (LZRR). (D) Cells transfected with PR and PD will begin in an “OFF” state since the PR is unphosphorylated and thus the GFP halves are detached. In the process of activation, (Di) the leucine zipper on the CDZK will recruit the leucine zipper on the PR, (Dii) the kinase will phosphorylate the Tyr residues on the PR, (Diii) the SH2 domain on the PD will bind to the phosphorylated Tyr on the PR, resulting in (Div) merging of the two GFP halves and an activated, “ON” state. (E) To induce deactivation, the CDZK is substituted with the constitutively dimerized zipper and phosphatase (ZPC) which contains the same leucine zipper as the CDZK, and a PTP 1B phosphatase domain in lieu of a kinase domain. (F) Cytosolic platform deactivation workflow. (Fi) The ZPC will bind to an activated PD/PR complex and remove the phosphoryl group from the PR, causing (Fii) the dissociation of two GFP halves, reverting the cell back to an “OFF” state.

### 2.2 Peptide-Ligand and Peptide-Functionalized Hydrogel Design

HA (sequence: NH_2_–YPYDVPDDYA-COOH), FLAG (sequence: NH_2_-DYKDDDDK-COOH), and MYC (sequence: NH_2_-EQKLISEEDL-COOH) peptide epitopes were selected as ligands that target candidate monobody receptors (Figure 2A). These epitopes are short and can be easily synthesized using solid state peptide synthesis and are generally considered orthogonal to cell components when used as tags in protein isolation and targeting experimentation using antibodies.^41,59^ Duplicated peptides were designed to have a matching epitope on each end (Figure 2B). This allows them to bind two of the same monobodies on two receptors for duplicated peptide screening experiments. The first iteration of these tests included a short, flexible five AA glycine/serine linker (GGSGG) between epitopes.^60^ No significant platform activation was found using these epitope ligands (data not shown), most likely from receptor-epitope steric hindrance. This occurs when a receptor, after binding to an epitope at one end of the duplicated peptide, physically obstructs another receptor from accessing the epitope at the other end of the peptide. To increase separation between the epitopes in our original duplicated peptide design, the next iteration of epitope tag ligands had a longer, 10 AA linker (GGSGG)_2_. For the FLAG–FLAG duplicated peptide-ligand, a slightly stiffer, but longer GSGSGSGSEAAAK linker was chosen due to its improved solubility and to minimize the potential of aspartimide formation during peptide synthesis which can impact its overall structure.^61,62^ After potential receptors were determined and extracellular linkers were optimized, the peptides were redesigned to contain two different epitopes on either end to create stimulatory and inhibitory peptide ligands. Additionally, a cysteine (‘c’) AA was placed in the middle of the linkers for thiol-norbornene peptide coupling to the hydrogel network (Figure 2C). The stimulatory peptide ligands contain HA and FLAG epitopes, and the inhibitory peptide ligands consist of HA and MYC epitopes. Taken together, the stimulatory HA-c-FLAG peptide ligand sequence used for heterodimer experiments is NH_2_-DYKDDDDKGGSGGCGGSGGYPYDVPDDYA-COOH and the inhibitory HA-c-MYC sequence is NH_2_-EQKLISEEDLGGSGGCGGSGGYPYDVPDYA-COOH. For determining the patterned area within hydrogels, a rhodamine molecule was attached to a thiolated peptide as previously described.^40^ Successful peptide synthesis for duplicated, stimulatory, inhibitory, and rhodamine-labeled peptides was confirmed using MALDI-TOF (matrix-assisted laser desorption/ionization-time-of-flight) spectroscopy (Supplemental Figure S1).

**Figure 2:**
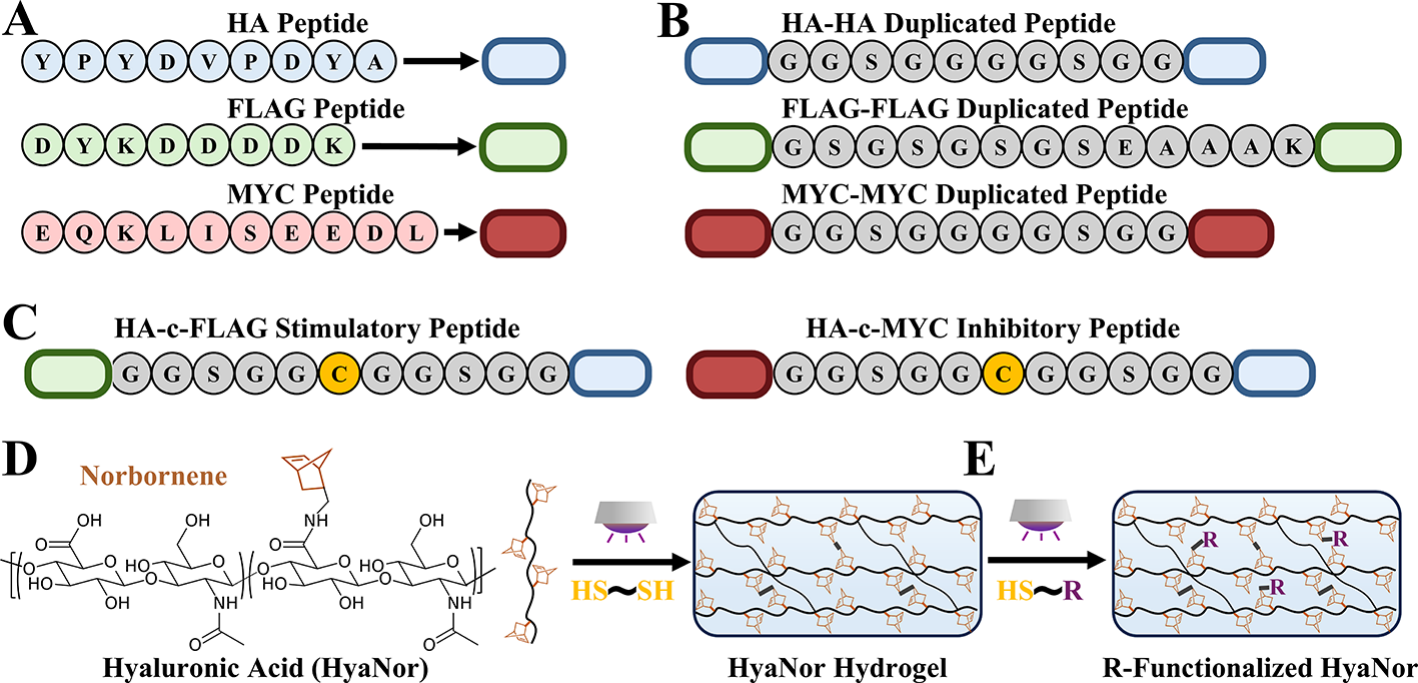
**Peptide-ligand and thiol-norbornene HyaNor hydrogel design.** (A) Graphical representation of HA, FLAG, and MYC epitope peptides. HA peptide (sequence: YPYDVPDYA) is denoted by a blue oval, FLAG peptide (sequence: DYKDDDDK) is denoted by a green oval, and MYC peptide (sequence: EQKLISEEDL) is denoted by a red oval. (B) Graphical representation of HA-HA, FLAG–FLAG, and MYC-MYC duplicated peptide designs used for peptide-monobody affinity screening and extracellular receptor linker length optimization studies. (C) Graphical representation of stimulatory HA-c-FLAG and inhibitory HA-c-MYC peptide-ligand heterodimer designs. These include a central thiol-containing cysteine (yellow) so that these peptides can be covalently bound to thiol-norbornene HyaNor hydrogels. (D) To form hydrogels, HyaNor macromers react with dithiolated cross-linkers, and (E) unreacted norbornenes in the hydrogel network can undergo a second reaction with monothiolated epitope peptides to covalently bind stimulatory (HA-c-FLAG) and inhibitory (HA-c-MYC) peptide heterodimers with spatial and temporal control.

To investigate EPDA receptor activation within 3D materials, biocompatible hydrogels amenable to spatial peptide functionalization were used. Hyaluronic acid macromers were modified with norbornene (HyaNor), and hydrogels were formed by reacting norbornene in HyaNor with dithiolated cross-linker peptides (Figure 2D). Peptide functionalization was achieved by reacting pendant norbornenes in HyaNor hydrogels with monothiolated peptides, resulting in peptide patterning only in regions exposed to light (Figure 2E). Dithiolated cross-linker peptide synthesis was confirmed using MALDI-TOF (Supplemental Figure S2), and synthesized HyaNor macromer had ∼45% of its repeat units functionalized with norbornene, as confirmed with ^1^H NMR (proton nuclear magnetic resonance) spectroscopy (Supplemental Figure S3). To demonstrate that HyaNor hydrogel peptide-functionalization does not affect mechanics, HyaNor hydrogels were formed without peptides or with HA-c-FLAG (100 μM), HA-c-MYC (100 μM), or thiolated rhodamine (100 μM) peptides and the elastic moduli were measured. Collectively, these hydrogels had an average elastic modulus of 4.8 kPa and no significant differences were observed between the groups (Supplemental Figure S4A). Since unreacted norbornenes in HyaNor hydrogels can undergo a second reaction with monothiolated peptide-ligands,^26^ we leveraged this chemistry to covalently bind stimulatory and inhibitory peptide heterodimers with spatial and temporal control. To show that these peptides could be effectively tethered to HyaNor hydrogels, a thiolated HA-c-FLAG peptide was synthesized to include a carboxyfluorescein molecule which fluoresces green (sequence: carboxyfluorescein-DYKDDDDKGGSGGYPYDVPDYAGGGCG-COOH). HyaNor hydrogels were exposed to a solution of fluorescent HA-c-FLAG peptides, and photopatterned using a photomask (vertical stripes, 50 μm width, 60 μm spacing) and UV light (5 mW/cm^2^, 5 min). Confocal microscopy images confirmed that HA-c-FLAG peptide-ligands were patterned onto HyaNor hydrogels only in regions that were exposed to UV light (Supplemental Figure S4B). The peptides described in this section will be used throughout this work to initiate the dimerization of two transmembrane receptors and activate the internal components described in Section 2.1. Monobodies were selected as the binding region due to their small size and potential to generate a variety of thermodynamically favorable interactions with these peptide ligands. A bottom-up design approach including the development of a computational design algorithm, described in the upcoming section, was taken to generate reliable and potent peptide binders which we could openly provide the sequences for.

### 2.3 PETEI Algorithm

After designing and characterizing peptide-ligands, we developed and used PETEI (Proteins Designed with Targeted Exceptional Interactions), a novel algorithm capable of identifying candidate monobodies based on epitope binding affinities. PETEI is motivated by the observation that protein binding interactions are commonly understood to have hotspots,^63^ where a hotspot is an AA that contributes a disproportionately large percentage of the overall binding energy. We hypothesized that monobodies designed to have strong hotspot interactions without large steric clashes or too many charged residues have a high likelihood of binding to target peptides when experimentally tested.

Monobodies are based on the 10th type III fibronectin domain, which is structurally similar to the antigen-binding variable domains of antibodies. Like antibodies, monobodies bind to targets through modular loops that can be exchanged to alter binding affinities. The three binding loops of monobodies are attached to a scaffold that comprises the rest of the monobody domain and is unaltered between different designs. PETEI computationally predicts monobodies that feature multiple, excellent thermodynamic binding interactions by generating a database of modular parts followed by selecting loops from those parts that bind to peptides of interest.

Chain A from Protein Data Bank^64^ (PDB) file 1TTG^65^ was selected as the scaffold for the monobody designs. The three binding loops designed by PETEI replace residues 22–30, 51–55, and 76–87 in file 1TTG. The generated database had 707 structures for loop 1, 708 for loop 2, and 958 for loop 3, for a total combinatorial diversity of approximately 480 million possible monobodies and was completed in 4 h and 53 min on a single processor of Auburn University’s Hopper Cluster. This database of loops is provided in Supplemental Workbook 1 and was used to generate monobody designs for HA, FLAG, and MYC peptides. Residues 58–65 of chain D from PDB file4WE7were used as the structure of the HA peptide,^66^ all seven residues of chain C from PDB file5CXVwere used as the FLAG peptide,^67^ and residue 3–31 of chain A from PDB file 1A93 were used as the MYC peptide.^68^ PETEI predicted 372 HA-binding monobodies in 1 h and 55 min, 1,110 FLAG-binding monobodies in 5 h and 42 min, and 582 MYC-binding monobodies in 8 h and 3 min. Each set of calculations was performed using a single processor on Auburn University’s Hopper Cluster. PDB files of the predicted complexes are available in the Supplemental Zip 1. The binding loops of the predicted monobodies have minimal similarity to those of the structure in 1TTG, with an average sequence similarity of 7.19%, which is consistent with what would be expected by random chance.

These complexes were then minimized, and their predicted binding energies and buried surface areas were calculated. The candidate monobodies were ranked for experimental testing using their predicted binding energy per buried surface area (BE/BSA), and the distributions for the PETEI-predicted HA (n = 372), FLAG (n = 1,110), and MYC (n = 582) binding monobodies are shown in Figure 3. For comparison, the same data for a nonredundant database of antibody-protein complexes (n = 231) is included.^69^ Antibodies are used as a comparison because they are the archetypical binding protein and there are far more experimentally determined antibody-protein complexes than those of monobodies. Collectively, FLAG-binding monobodies have significantly better values (p = 0.0053), HA-binding monobodies have comparable values (p = 0.1735), and MYC-binding monobodies have significantly lower values (p = 4.7 × 10^–11^) than the naturally occurring antibodies, where significance was assessed using two-tailed *t* tests assuming unequal variances. From these, the 10 highest ranking monobody designs for each peptide were selected for experimental testing (Table S1). Although prior computational methods to design antibodies had used predicted binding energies as the criteria to select structures for experimental testing,^46,48^ BE/BSA was used here. This is because a plot of binding energies versus buried surface areas for the 231 antibody-protein complexes shows a clear correlation between the two values (Supplemental Figure 5). While this is consistent with prior computational studies, it is inconsistent with known experimental results.^70^ Therefore, BE/BSA was used as the criteria to select designs for experimental testing in this study.

**Figure 3:**
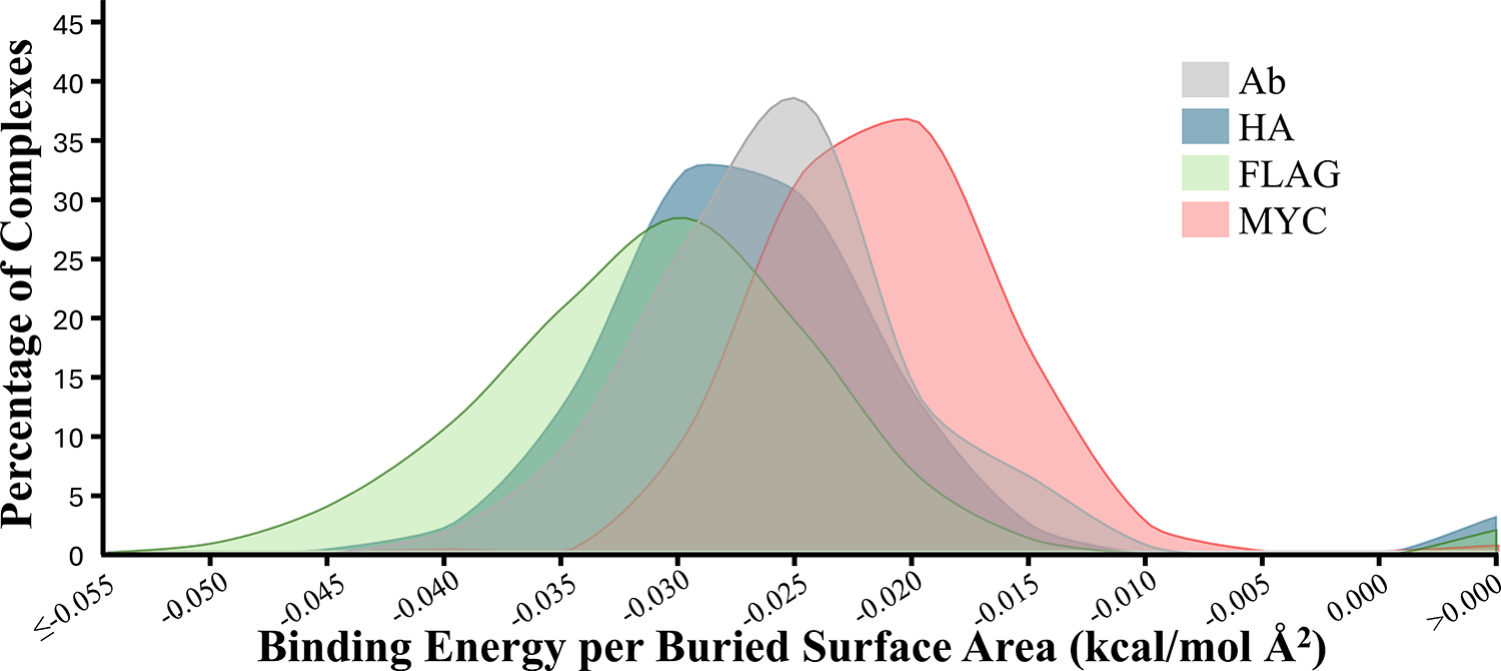
**Binding energies per buried surface area for predicted monobodies.** Histogram of the binding energies per buried surface area for PETEI-predicted HA (blue), FLAG (green), and MYC (red) binding monobodies, and naturally occurring antibodies from a nonredundant antibody-protein database (Ab, gray). The FLAG-binding monobodies have significantly better values (*p* = 0.0053), the HA-binding monobodies have comparable values (*p* = 0.1735), and the MYC-binding monobodies have significantly worse values (*p* = 4.7 × 10^–11^) than naturally occurring antibodies, where significance was assessed using two-tailed *t* tests assuming unequal variances.

### 2.4 HA, FLAG, and MYC Monobody Receptor Screening and Selection

To test candidate monobodies designed with our PETEI algorithm, a push–pull post translational phosphorylation circuit^49^ was adapted for use in a transmembrane receptor dimerization platform to determine the potential for monobody receptors to respond to extracellular ligand inputs. Often antigen targeting tools like scFvs which were developed for direct antigen targeting are unsuccessful as transmembrane receptors due to problems like steric hindrance, surface aggregation, and surface expression. To avoid these complications, we chose to screen these monobodies directly in their intended application of a transmembrane receptor rather than using other approaches like ELISA (enzyme-linked immunosorbent assay) or SPR (surface plasmon resonance). The intracellular components of the receptor utilize the LZRR to recruit the phosphorylation recipient to one-half of the receptor. The intracellular region of the other half of the receptor includes the ABL-I kinase. Once phosphorylated, the tyrosine domains recruit the phosphorylation detector which is monitored using green fluorescence. The kinase receptor domain on the stimulatory receptor can be replaced with a phosphatase which can selectively remove the phosphoryl group on CD3ζ, resulting in fluorescent protein dissociation and platform inactivation. This platform relies on inducing downstream protein dimerization rather than altering fluorescent protein fluorescence emission through transcription as is the case with many transmembrane receptor systems. Because of this we regard statistically significant increases in mean fluorescent intensity as a potent response at the receptor level indicative of a successful peptide binder.

To minimize the number of initial combinations, and to isolate the effect of each monobody binder, the receptors were self-matched and duplicated peptides were used as ligands. FKBP and FRB rapamycin binding proteins,^71^ known to dimerize in response to rapamycin, were used to validate this design and determine a starting point for which extracellular linkers to use for zipper and kinase receptor halves. In this initial study, all groups showed an increase in fluorescence in response to the rapamycin ligand (Supplemental Figure S6). When the 20 AA linker was used on both the zipper and kinase halves, the cells exhibited the highest fold change, so 20 AA linkers were used during the first screening monobody receptor screening round with the plan to later optimize their length.

The HA-HA duplicated peptide was used to examine the responsiveness of each PETEI-identified HA monobody, termed HA1 to HA10 (Figure 4A). Four of the ten computationally derived monobodies exhibited a small and insignificant fold change indicating insufficient dimerization of the transmembrane receptors. Two monobodies showed high background fluorescence indicating potential receptor aggregation or ligand independent dimerization, resulting in a lack of desired orthogonality and specificity. 40% of the HA monobodies did show a significant increase in fluorescence and encouragingly, the HA3 receptor showed low background fluorescence in the no peptide ligand group and a significant 1.92-fold increase in the HA-HA peptide ligand treatment group (Figure 4B). This screening process was repeated with the FLAG receptors, termed FLAG1 to FLAG10 (Figure 4C). Again, three of these computationally derived pairs showed limited effectiveness, and two showed minimal fold changes due to high background dimerization. 50% of the monobodies showed a significant increase in fluorescence and the FLAG5 receptor exhibited a 2.61-fold increase between the no ligand and peptide ligand groups and was used for further optimization (Figure 4D). The same receptor screening exercise was performed with computationally derived monobody receptors specific to the MYC epitope tag, termed MYC1 to MYC10, using MYC-MYC duplicated peptides (Figure 4E**)**. Only the MYC7 receptor showed a significant increase in fluorescence, with an increase of 34% between Peptide (−) and Peptide (+) groups (Figure 4F).

**Figure 4:**
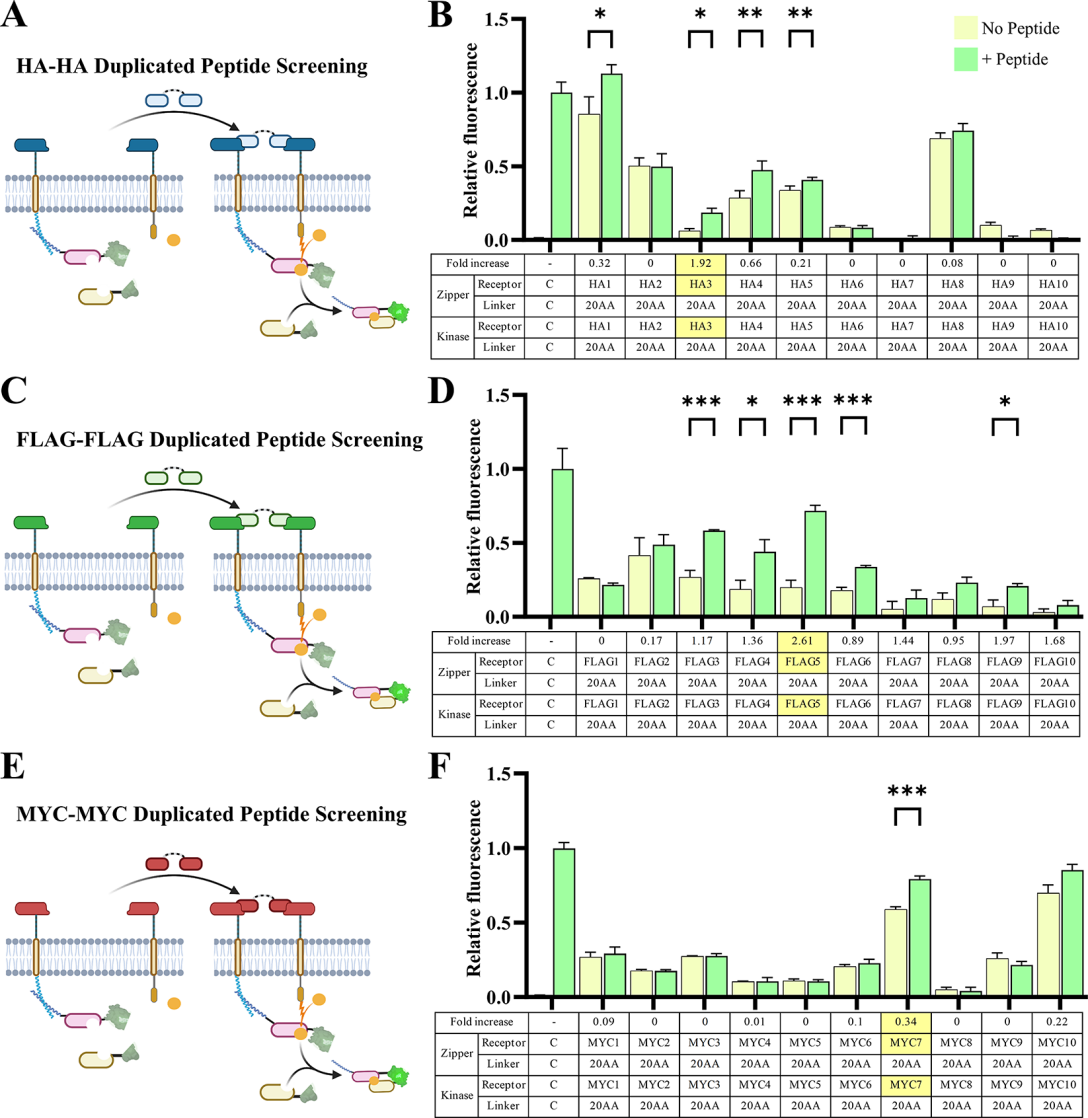
**Experimental screening identifies monobodies that are most responsive to HA, FLAG, and MYC peptides.** (A) Workflow of extracellular HA-HA duplicated peptides activating intracellular GFP merging via HA peptide interactions with candidate HA monobodies. (B) HA3 monobody shows the highest increase (192%) in green fluorescence between the absence and presence of HA-HA peptide. (C) Workflow of extracellular FLAG–FLAG duplicated peptides activating intracellular GFP merging via FLAG peptide interactions with candidate FLAG monobodies. (D) FLAG5 monobody shows the highest increase (261%) in green fluorescence upon FLAG–FLAG peptide activation. (E) Workflow of extracellular MYC-MYC duplicated peptides activating intracellular GFP merging via MYC peptide interactions with candidate MYC monobodies. (F) MYC7 monobody shows the highest increase (34%) in green fluorescence between the absence and presence of MYC-MYC peptide. Fluorescence was normalized by setting the PR and PD negative control fluorescence as 0 and the PR, PD, and CDZK positive control fluorescence as 1. Error bars represent standard error around the mean (s.e.m.); * *p* < 0.05, ** *p* < 0.01, *** *p* < 0.001; *n* = 4 for each group, with at least 10,000 cells assayed per Peptide (−) and Peptide (+) treatment via flow cytometry.

In these experiments, we were able to take the hypothesized monobody sequences derived *in silico* and screen them *in vitro* (Figure 4). The computational model of these three selected monobodies (HA3, FLAG5, and MYC7) have multiple AAs within the binding loop regions which are within 5 Å of the peptides of interest (Supplemental Figure S7). This suggests strong molecular interactions which are believed to drive the binding seen experimentally. A particularly notable finding comes from comparing the distributions of BE/BSA to the experimental properties of the tested monobodies. The FLAG-binding monobodies had the best distribution of BE/BSA, followed by the HA-binders, with the MYC-binders having the worst distribution (Figure 3). The FLAG-binding monobodies had both the most experimental hits and the largest fold changes in fluorescence, while the HA-binders had fewer experimental successes, and only one of the MYC-binding monobodies was experimentally viable.

To the author’s knowledge, no prior study that has tested at least 30 computationally designed proteins achieved a 33% success rate as shown here. The best success rate that we are aware of was by RFdiffusion, which had fewer than 20% successful experimental binders.^72^ We hypothesize that this success rate is due to PETEI’s approach to designing binding proteins, which is ensuring designs have enough exceptional interactions and no obviously detrimental features. We intend to evaluate this hypothesis in future work by experimentally testing with many very good interactions, no obvious detrimental features, and affinity maturation mutations. We will also retrospectively study the numbers of very good interactions in prior experimentally successful and unsuccessful computationally predicted binding proteins. It is noted that this *in vitro* approach to verifying protein–protein binding cannot be directly compared to other methods. Also, while the 100 μM concentration of peptide used for screening is appropriate for the EPDA platform, other biochemical assays may have a higher success rate if other ligand concentrations are utilized. As machine-learning based methods continue to proliferate, the ability to design proteins that have certain features or look certain ways will become increasingly simpler. The inclusion of rationale, explainable features, in those algorithms such as targeted, thermodynamically exceptional interactions as PETEI does, may be a pathway to rapidly designing proteins with a high rate of experimental success.

### 2.5 HA, FLAG, and MYC Receptor Extracellular Linker Optimization

Flexible intracellular or extracellular linkers can be used to reduce steric hindrance, and linker optimization is an important consideration during the developmental phase of new transmembrane receptors.^73^ Once binding domains that minimized off target phosphorylation and had a high fold change above this baseline were determined, optimal external linker lengths were determined, again using this receptor matched experiment and duplicated peptides for receptor homodimerization. For both the HA3 kinase and zipper receptors, linkers which contained the flexible serine, glycine and threonine AAs^61^ with sequences of 10 (TSGTGGSGTG), 20 (T­(SGGTGG)_2_(SG)_2_GTG)), and 30 (TSGTGGSGAGTGGSAGGTGGSGGGGGSA) AAs in length were generated, and all nine combinations of these receptor pairs were tested (Figure 5A). It was shown that the HA3 receptor with a 10 AA extracellular linker was responsive to ligand activation no matter the kinase linker length. Because of this, the HA3 receptor including a 10 AA extracellular linker, and an internal leucine zipper (termed H3ZA) would be utilized for heterodimer experiments (Figure 5B). Of note, the HA3 20 AA zipper linker and 20 AA kinase linker that was used in both experiments did decrease from 192% to 79% but maintained a significant increase in GFP fluorescence when reanalyzed (*p* < 0.001).

**Figure 5:**
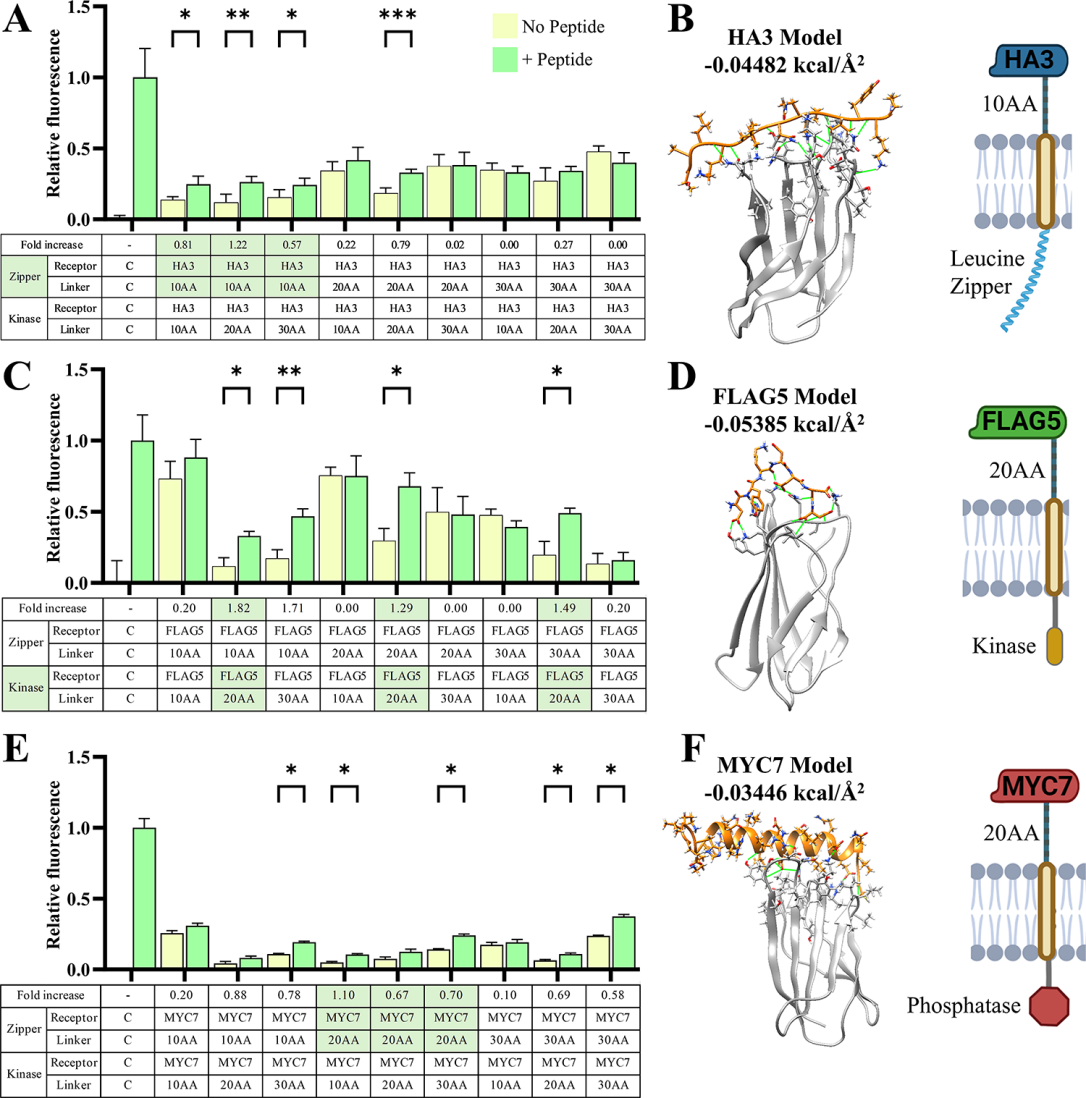
**Experimental screening identifies optimal extracellular zipper, kinase, and phosphatase linker lengths.** (A) The HA3 monobody attached to the leucine zipper intracellular domain and a 10 AA extracellular linker length (termed H3ZA) showed the highest fold change (1.22) in green fluorescence upon HA-HA duplicated peptide activation. (B) Rosetta computational model of the HA3 monobody binding to the HA peptide has a predicted BE/BSA of −0.045 kcal/Å^2^. (C) The FLAG5 monobody attached to the kinase intracellular domain and a 20 AA extracellular linker length (termed F5KB) showed the highest fold change (1.82) in green fluorescence upon FLAG–FLAG duplicated peptide activation. (D) Rosetta computational model of the FLAG5 monobody binding to the FLAG peptide has a predicted BE/BSA of −0.054 kcal/Å^2^. (E) The MYC7 monobody attached to the leucine zipper intracellular domain and a 20 AA extracellular linker length (termed M7PB) showed the highest fold change (1.10) in green fluorescence upon MYC-MYC duplicated peptide activation. (F) Rosetta computational model of the MYC7 monobody binding to the MYC peptide has a predicted BE/BSA of −0.034 kcal/Å^2^. Error bars represent standard error around the mean (s.e.m.); * *p* < 0.05, ** *p* < 0.01, *** *p* < 0.001; *n* = 4 for each group, with at least 10,000 cells assayed per Peptide (−) and Peptide (+) treatment via flow cytometry.

This linker optimization experiment was repeated with the FLAG5 receptor (Figure 5C). It was determined that when the FLAG5 receptor included an internal kinase and 20 AA extracellular linker (termed F5KB), it showed significantly higher emission of green fluorescence after ligand treatment across all zipper halves (Figure 5D). F5KB exhibited the highest fold change in response to peptide in the presence of a zipper receptor containing a 10 AA linker. This is beneficial because the best performing HA receptor, H3ZA, contains a 10 AA linker and should perform well when used in combination with F5KB. For these reasons F5KB and H3ZA were selected for use in combination for future experiments. Historically, *de novo*, computationally derived protein binders have a success rate in the ranges or below the results presented by these monobodies.^72^ The optimal linker length of MYC7 was also tested in the same manner (Figure 5E). It was determined that this receptor worked best when a 20 AA was attached to the zipper or kinase which showed average fold increases of 82% and 72% respectively, across linker lengths (Figure 5F). The MYC7 receptor was attached to an intracellular phosphatase (termed M7PB) to be used for ligand responsive platform deactivation. The surface expression of H3ZA, F5KB, and M7PB receptors was confirmed using flow cytometry by tagging and staining them with fluorescent antibodies without permeabilization (Supplemental Figure S8). In summary, the H3ZA and F5KB receptors are used for the Stimulatory EPDA design, which activates intracellular phosphorylation in response to the HA-c-FLAG peptide, and the M7PB receptor is included in the Inhibitory EPDA design to dimerize with the H3ZA receptor in response to the HA-c-MYC peptide which removes this phosphoryl group to deactivate the platform.

### 2.6 Stimulatory EPDA Receptors Sense and Respond to HA-c-FLAG Peptide-Ligands

After HA zipper (H3ZA) and FLAG kinase (F5KB) receptors were identified and optimized independently, they were used in conjunction with each other to create Stimulatory EPDA receptors (Figure 6A). This design was chosen to maximize the probability that a kinase and zipper are brought together in response to an activating peptide-ligand rather than from unproductive dimers of the same receptor type, as seen previously.^31^ The peptide-ligand consists of an HA epitope and a FLAG epitope separated by an 11x AA linker which includes a central cysteine AA. As expected, upon peptide-ligand activation, the receptors functioned as dimerization actuators, transmitted phosphorylation to the tyrosine groups, and promoted SH2 dimerization and split GFP molecule reconstitution, as evidenced by a significant increase in green fluorescence between peptide-ligand and peptide-free groups (Figure 6B). The mean fluorescent intensities of the samples from this experiment before normalization are shown in Supplemental Figure S9. These Stimulatory EPDA receptors demonstrate the successful translation of an *in-silico* screening process into a functional transmembrane receptor construct. This peptide-ligand receptor system is the first of its kind, and the process that led to its development can be applied to a plethora of *de novo* peptide receptors. Being derived from natural AAs, peptide-ligands are biocompatible and can be incorporated to a myriad of biomaterials with spatial and temporal control. Specifically, the inclusion of a central cysteine in our HA-c-FLAG peptide-ligand design enables covalent coupling of the peptide into HyaNor hydrogels via light-mediated thiol-norbornene click reactions.

**Figure 6:**
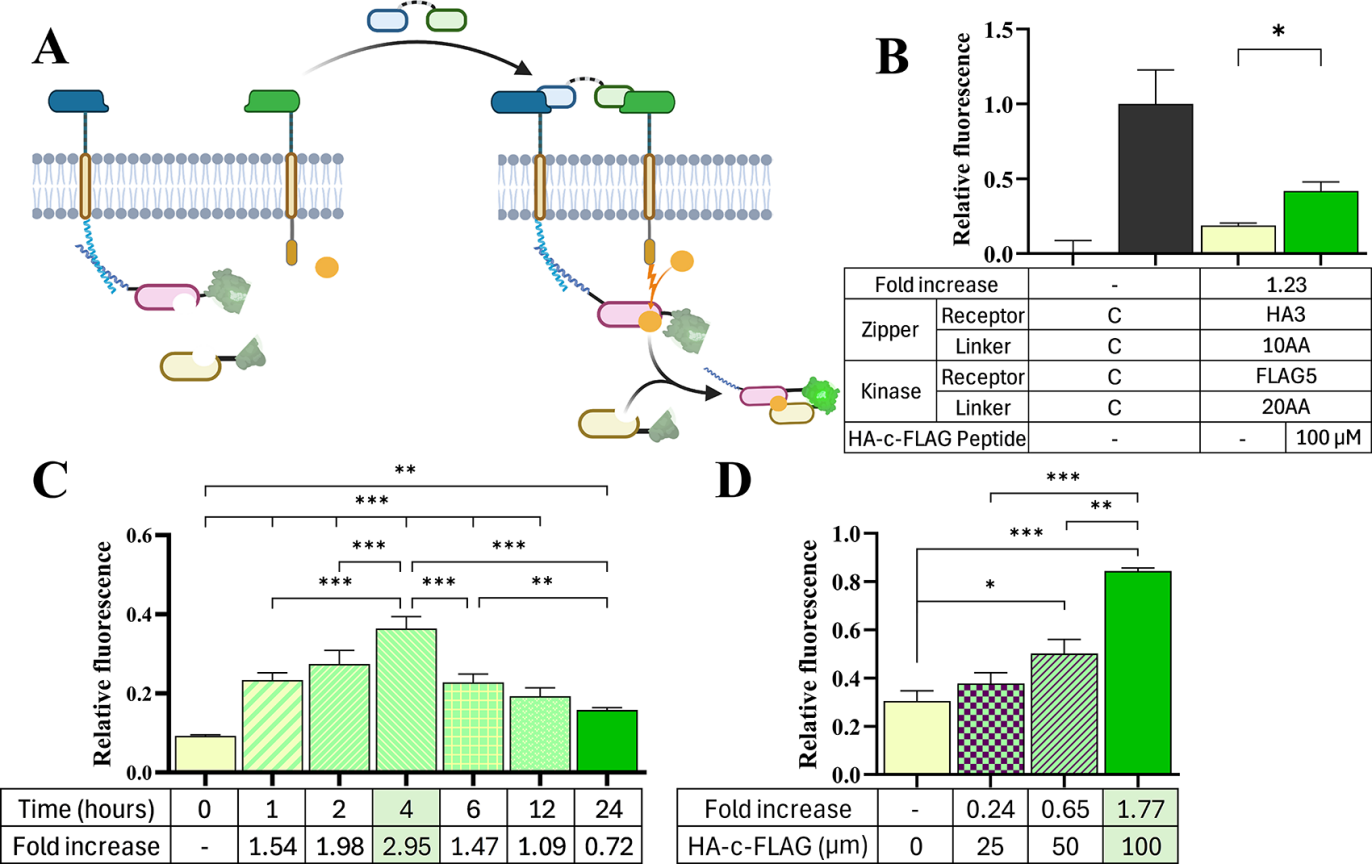
**Effects of HA-c-FLAG peptide heterodimer timing and dosing on activating Stimulatory EPDA receptors.** (A) Schematic of extracellular HA-c-FLAG peptide heterodimer activating intracellular GFP merging via HA3 and FLAG5 interactions with corresponding zipper and kinase monobodies in a Stimulatory EPDA receptor. (B) HA-c-FLAG peptide (100 μM) activation induced a significant increase (123%) in green fluorescence in comparison to fluorescence in the absence of peptide after 24 h of soluble peptide ligand exposure. To determine HA-c-FLAG peptide activation as a function of time, (C) transfected HEK293 cells were treated with HA-c-FLAG peptides (100 μM) and subject to flow cytometry at 1, 2, 4, 6, 12, and 24 h post peptide-ligand treatment. The highest increase in green fluorescence (295% between peptide and nonpeptide groups) was observed at 4 h post-treatment. To determine HA-c-FLAG peptide activation as a function of peptide-ligand concentration, (D) transfected HEK293 cells were treated with HA-c-FLAG peptides at 0, 25, 50, or 100 μM and subject to flow cytometry at 1 h post peptide-ligand treatment to highlight the system’s rapid response time. The highest increase in green fluorescence (177% between peptide and nonpeptide groups) was observed at a 100 μM concentration. Error bars represent standard error around the mean (s.e.m.); * *p* < 0.05, ** *p* < 0.01, *** *p* < 0.001; *n* = 4 for each group, with at least 10,000 cells assayed per Peptide (−) and Peptide (+) treatment via flow cytometry.

Evaluation of peptide-ligand induced activation of EPDA receptor components was performed 24 h post peptide-ligand exposure, and we were interested in investigating the response dynamics of this novel heterodimer receptor construct. It was determined that after just 1 h post peptide-ligand exposure, cells transfected with Stimulatory EPDA receptors displayed a significant increase in split fluorescence reporter emission, with increasing fluorescence which peaked at 4 h (Figure 6C). It is possible that decreased activation after a rapid maximal fluorescence at 4 h is the result of the soluble peptides being endocytosed or processed by the cells. However, we attribute the bulk of the decrease to the reversibility of this platform and the ZPC included in receptor experiments. The 24-h time point used for previous investigations was lower than this 4 h time point but still showed a significant increase in fluorescence over the no treatment group. Phosphorylation and split fluorescent refolding and maturation have been shown to have much faster response rates than the transcription and translation of a fluorescent reporter.^32,35,50^ This rapid detection capability allows for a better understanding of the dynamics of this new ligand type and is one of the primary motivations for utilizing this phosphorylation circuit and split fluorescence as a readout.

Peptide-ligand dosing was done at the shortest time point (1-h) previously shown to exhibit a significant fold change post peptide-ligand exposure. The HA-c-FLAG peptide showed a significant increase in platform activation at the 50 μM concentration with a fold change of 65% and this increased to 177% at a concentration of 100 μM (Figure 6D). Representative histograms of raw green fluorescence data for relative green fluorescence data are shown in Supplemental Figure S10. Previous experimentation showed that peptide concentrations of 200 μM and above affected cell health, whereas cells treated with 100 μM for 36 h had no effect on transfected cell viability, so this was chosen as the upper dosing limit (Supplemental Figure S11). This range of concentrations indicates that at 50 μM this platform can be activated but that there are still some unbound receptors. This can be useful for future experimentation with multiple peptide ligands. The selectivity to HA-c-FLAG peptide-ligands was evaluated by treating Stimulatory EPDA-transfected cells with a scrambled version of HA-c-FLAG (NH_2_–KDDKDYDDGGSGGCGGSGGYAYYVPDP-COOH), HA-c-MYC peptide, and a full length TGF-β dimer. As expected, these ligands did not induce a change in relative GFP fluorescence, whereas exposure to the stimulatory HA-c-FLAG peptide-ligand resulted in a significant increase in GFP fluorescence (Supplemental Figure S12). Of note the peptide-treated groups (solid green bar) shown in Figures 6 B-D and Supplemental Figure 12 received the same treatment of 100 μM of HA-c-FLAG peptide for 24 h, which resulted in a consistent and reproducible increase in green fluorescence compared to the no ligand group within the same experiment. This stimulatory platform can activate in response to soluble peptide in a rapid and dose dependent manner. The trend that the peptide induced fluorescence is reduced from its maximum after 4 h is likely driven by the inclusion of the phosphatase on the ZPC. The phosphatase was placed on the MYC7 receptor to investigate this idea and determine if this reduction in fluorescence can be induced by an extracellular peptide ligand.

### 2.7 Inhibitory EPDA Receptors Reverse Stimulatory EPDA Receptor Activation

After Stimulatory EPDA receptor activation dynamics in response to HA-c-FLAG peptides were established, we were interested in investigating if Inhibitory EPDA receptors can reverse this response in the presence of HA-c-MYC peptides. Boolean logic gates can enhance safety and efficacy of cell-based systems and have been used in the fields of biomaterials and synthetic biology to increase control over sense-and-respond mechanisms.^6^ To evaluate the ability to reversibly control split GFP merging, a NIMPLY Boolean logic gate was developed which activates GFP fluorescence only in the presence of HA-c-FLAG, and shows a lack of activation in the following scenarios: no peptide, inhibitory HA-c-MYC peptide, or in the presence of stimulatory and inhibitory peptides at equimolar concentrations (Figure 7A). In this mechanism, HA-c-FLAG peptide-ligands cause H3ZA and F5KB Stimulatory EPDA receptor dimerization and subsequent phosphorylation of the internal substrate and green fluorescence (Figure 7B). The addition of HA-c-MYC peptide-ligands activate Inhibitory EPDA receptors by causing H3ZA dimerization with the phosphatase receptor, M7PB (Figure 7C). This will cause the dephosphorylation of any background level phosphorylation and an expected small decrease in fluorescence. In the presence of both peptide ligands, the HA epitope on HA-c-FLAG and HA-c-MYC peptides can bind to the H3ZA receptors, and the phosphatase and kinase receptors will be recruited to substrates to add and remove phosphoryl groups, respectively.

**Figure 7:**
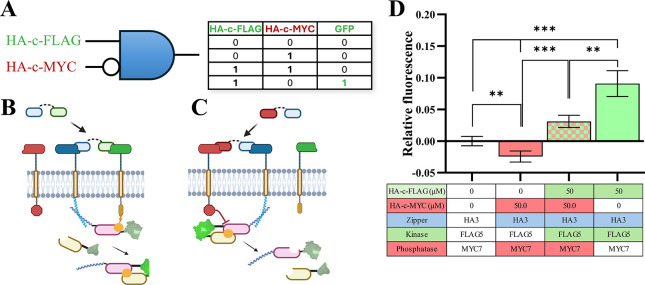
**Evaluating Stimulatory and Inhibitory EPDA receptor dynamics using Boolean logic gates.** (A) Overview of EDPA receptor NIMPLY Boolean logic gate. GFP fluorescence is only achieved in the presence of stimulatory HA-c-FLAG peptide, and in the absence of peptide or in the presence of inhibitory HA-c-MYC or equimolar concentrations of stimulatory and inhibitory peptides no fluorescence is observed. (B) The Stimulatory EPDA receptor system features an HZ3A zipper recruiter receptor and a FK5B kinase activator receptor that adds a phosphoryl group in the presence of HA-c-FLAG peptide (split GFP merging). (C) The Inhibitory EPDA receptor systems feature an HZ3A zipper recruiter receptor and an M7PB phosphatase inhibitory receptor that removes a phosphoryl group in the presence of HA-c-MYC peptide (split GFP dissociation). (D) Relative green fluorescence plot shows minimal activation in the absence of peptide, in the presence of 50 μM HA-c-MYC peptide. In contrast, in the presence of stimulatory HA-c-FLAG peptide (50 μM) there is an approximately 2-fold increase in fluorescence over the equimolar treatment. Error bars represent standard error around the mean (s.e.m.); ** *p* < 0.01, *** *p* < 0.001, *n* = 4 for each group, with at least 10,000 cells assayed per treatment group via flow cytometry.

As expected, cells transfected with Stimulatory and Inhibitory EPDA receptors exhibit baseline GFP fluorescence, and exposure to 50 μM of inhibitory HA-c-MYC peptide led to a reduction in fluorescence below peptide-free levels. At an equimolar peptide-ligand dose of stimulatory and inhibitory peptides (50 μM each), there is a significant increase in GFP fluorescence when compared to peptide-free and HA-c-MYC peptide groups. In this scenario, GFP relative fluorescence was 0.03, which is significantly lower than fluorescence in the presence of stimulatory peptide (50 μM) alone (Figure 7D). This suggests that in the presence of the inhibitory peptide ligand the phosphatase receptor can reverse any background phosphorylation events and is effective at inhibiting platform activation. The mean fluorescent intensities of the samples from this experiment before normalization are shown in Supplemental Figure S13. To further highlight the ability to reversibly control GFP fluorescence, we induced GFP fluorescence using stimulatory peptides and evaluated GFP fluorescence deactivation using inhibitory peptides (Supplemental Figure S14). Cells exposed to stimulatory peptides for 24 h had higher GFP relative fluorescence than those exposed to inhibitory peptides for 24 h, and cells exposed to stimulatory peptides for 20 h exhibited a complete abrogation in fluorescence upon exposure to inhibitory peptides for 4 h (hours 20–24). Together, this demonstrates that the inhibitory platform works in both a concentration and time-dependent manner. While these results consistently show a response to stimulatory HA-c-FLAG peptide ligand, the addition of the receptor phosphatase tends to reduce the magnitude of the response to the stimulatory peptide relative to the controls (Figure 7) as compared to the results seen with no M7PB present (Figure 6). We attribute this to the phosphatase being localized at the surface and removing phosphoryl groups from the PR, thereby reducing split fluorescent protein reconstitution. Although receptor systems featuring inhibition through competitive extracellular binding have been previously developed,^74^ to our knowledge this is the first receptor system capable of controlling platform activation using stimulatory and inhibitory peptide-ligands.

### 2.8 Stimulatory EPDA Receptor Interactions with Peptide-Ligands in 3D HyaNor Hydrogels

After demonstrating that cells transfected with Stimulatory and Inhibitory EPDA receptors sense and respond to HA-c-FLAG and HA-c-MYC peptides in their soluble form, we were interested in evaluating the ability for cells programmed with EPDA receptors to sense and respond to covalently bound stimulatory HA-c-FLAG peptides in 3D materials. Here, we use HyaNor hydrogels formed by reacting HyaNor macromers with dithiolated cross-linker peptides and leverage pendant norbornene groups for spatial patterning of the hydrogels with monothiolated peptides. By following this two-step process, we demonstrated spatiotemporal peptide tethering by forming base HyaNor hydrogels that were later photopatterned with thiolated rhodamine peptides (Supplemental Figure S15). To demonstrate that peptides only bind to hydrogel regions exposed to light, we used a large “V_L_” Vega Lab logo photomask (7.0 mm × 5.8 mm) (Supplemental Figure S15A). Using this scheme, we also used a striped vertical mask (10 μm width, 40 μm spacing) to pattern features in the micrometer scale (Supplemental Figure S15B), and tethered peptide patterns retained their geometry and intensity for at least 100 μm into the hydrogel (Supplemental Figure S15C). Thus, HyaNor hydrogels are an excellent platform for exposing encapsulated cells to peptide-ligands in a 3D material environment.

HEK293 cells transfected with an eBFP (enhanced blue fluorescent protein) transfection control and Stimulatory and Inhibitory EPDA receptor components (PD, PR, H3ZA, and F5KB) were suspended in culture medium (5 million cells/ml) containing HyaNor macromer (3 wt %), photoinitiator (I2959, 0.05 wt %), dithiolated cross-linker peptide (3 mM), and adhesive thiolated RGD peptide (2 mM, sequence: GCGYGRGDSPG, MALDI-TOF spectra shown in Supplemental Figure S16), and the cell-laden hydrogel solution was placed in 8 mm cylindrical silicone molds and irradiated with UV light (6 mW/cm^2^, 5 min). 20 h post-encapsulation, cell-laden hydrogels were submerged in a solution of HA-c-FLAG peptide (100 μM) and thiolated-rhodamine peptide (100 μM) for 10 min, and a square photomask (230 μm × 230 μm) was used to pattern the stimulatory peptides and rhodamine to visualize patterns with UV light (6 mW/cm^2^, 5 min) (Figure 8A). HA-c-FLAG peptide patterning was successful and spanned across the entire hydrogel (Figure 8B), and cells were in and out of stimulatory regions (Figure 8C), allowing for imaging-based single-cell spatial analysis of GFP fluorescence (Figure 8D). Blue-fluorescent cells were transfected with Stimulatory EPDA receptors, and qualitatively EPDA receptor-modified cells inside stimulatory squares (Figure 8E) had higher green fluorescence per voxel volume than those in peptide-free regions (Figure 8F). Quantitatively, a significant (151%) increase in green fluorescence in EPDA-modified cells inside HA-c-FLAG dosed squares was observed when compared to cells outside of stimulatory regions (Figure 8G). This data is provided in terms of green fluorescence per μm^3^ in Supplemental Figure S17. Various angles of cells within a stimulatory square area fluorescing green and cells within the surrounding area exhibiting minimal fluorescence are shown in Supplemental Video 1 and representative images of this video are shown in Supplemental Figure S18.

**Figure 8:**
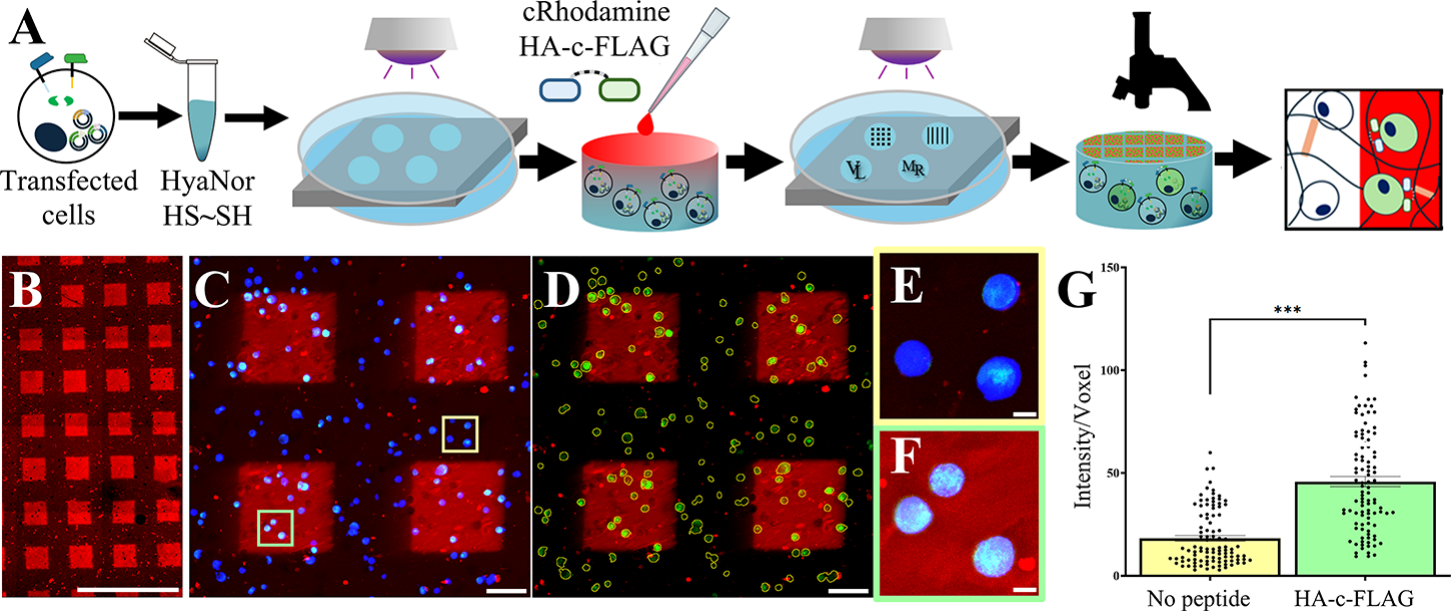
**Encapsulated Stimulatory EPDA-modified cells spatially respond to HA-c-FLAG activation.** (A) Schematic of transfected cell encapsulation and stimulatory peptide-ligand photopatterning workflow. Briefly, transfected cells are suspended in a hydrogel solution consisting of HyaNor macromer, photoinitiator, thiolated RGD peptide, and dithiolated cross-linker peptide, placed in silicone molds, and irradiated with UV light. 20 h post-gelation, cell-laden hydrogels are submerged in a solution of photoinitiator, HA-c-FLAG peptide, and thiolated rhodamine peptide (cRhodamine), and patterns are achieved using photomasks and UV light. 4 h post-patterning, hydrogels are imaged and single cell green fluorescence in and out of stimulatory regions is evaluated. (B) Macroscopic image showing a top-down view of a HyaNor hydrogel patterned with 230 μm side length rhodamine squares. (C) Gel subsection showing rhodamine squares (red), transfected cells (blue), and activated cells (green). (D) Gel subsection with blue channel removed and transfected cell bodies demarcated as thin yellow lines. Zoomed in subsection of macroscopic image with cells in either (E) no peptide (yellow square) or (F) HA-c-FLAG peptide treated area (green square). (G) Single cell analysis results of the green fluorescence intensity per voxel found within transfected cell volume outside (red) and outside (green) of stimulatory HA-c-FLAG peptide treated areas. Error bars: SEM, *n* > 100 per region, *p* < 0.001 (***). Scale bars: B = 1 mm, C–D = 100 μm, E-*F* = 10 μm.

HEK293 cells transfected with 700 ng of the empty plasmid remained highly viable throughout the hydrogel encapsulation and peptide patterning process (Supplemental Figure S19). It is also notable that the cell volume and sphericity of transfected cells inside and outside square regions was not statistically different (Supplemental Figure S20), suggesting that stimulatory peptides have no effect on cell morphology while selectively promoting peptide-ligand mediated cell activation. This study also shows that stimulatory peptide-ligands are effective when covalently bound to 3D hydrogels and can control EPDA-modified cell activation with spatial (via photopatterning) and temporal (via user-defined time of patterning) control.

### 2.9 Spatial Orthogonal Control over Transmembrane Receptor Signaling in Biomaterials

While peptide-laden hydrogels expose cells to biochemical signals that regulate cell behavior,^14^ these cell-material interactions are typically unidirectional and rely on native signaling pathways.^7^ After demonstrating that encapsulated cells transfected with Stimulatory EPDA receptors sense and respond to regions functionalized with stimulatory HA-c-FLAG peptides, we were interested in studying 3D cell-material interactions between cells transfected with Stimulatory and Inhibitory EPDA receptors and hydrogel-tethered HA-c-FLAG and HA-c-MYC peptide ligands. Cells transfected with Stimulatory and Inhibitory EPDA receptor components (H3ZA, F5KB, and M7PB) were suspended in a HyaNor hydrogel solution (HyaNor macromer, photoinitiator, dithiolated cross-linker peptide, and thiolated RGD peptide in culture media), which was transferred to a cylindrical mold and irradiated with UV light to form hydrogels. After 20 h, HyaNor hydrogels were soaked in a solution of 50 μM of HA-c-FLAG peptide and 100 μM thiolated rhodamine and irradiated with UV light in the presence of a square-containing (230 μm side length) photomask. Patterned hydrogels were thoroughly rinsed with PBS and then soaked in a solution of 50 μM HA-c-MYC peptide and 100 μM thiolated rhodamine and irradiated with UV light in the presence of a circle-containing (225 μm diameter) photomask (Figure 9A). The thiolated rhodamine was added to visualize the patterns, and inhibitory circle and stimulatory square regions were clearly visible throughout the hydrogel, indicating successful spatial tethering of inhibitory HA-c-MYC and stimulatory HA-c-FLAG peptides (Figure 9B). Cells in 3D square regions were visibly greener than those found outside of stimulatory peptide dosed areas, within circular inhibitory peptide sections, or where circular and square patterns overlapped (Figure 9C-L). This demonstrates that Stimulatory EPDA receptors sense and respond to HA-c-FLAG only in treated areas, and that Inhibitory EPDA receptors abrogate GFP fluorescence in inhibitory peptide regions. Quantitatively, a 106% increase in fluorescence in the HA-c-FLAG stimulatory peptide treated group was seen over the no peptide group and a significant difference between this stimulatory group was observed across all other groups (Figure 9M). It is also important to note that no significant differences in fluorescence were seen between no peptide, inhibitory peptide regions, and areas containing both stimulatory and inhibitory peptide ligands. This indicates that cells transfected with both Stimulatory and Inhibitory EPDA receptors are capable of exhibiting NIMPLY Boolean logic in response to material-laden peptides in a similar fashion as seen with these peptides in their soluble form (Figure 7). These ligands can be used in any number of biomaterial applications which allow for the spatial patterning of peptides onto biomaterials to produce even more sophisticated cell-material interactions.^4,75^ This platform allows for regulatory control over cell-material interactions and is expected to be a useful tool for various applications in investigational research and eventual cell-material based therapeutics.

**Figure 9:**
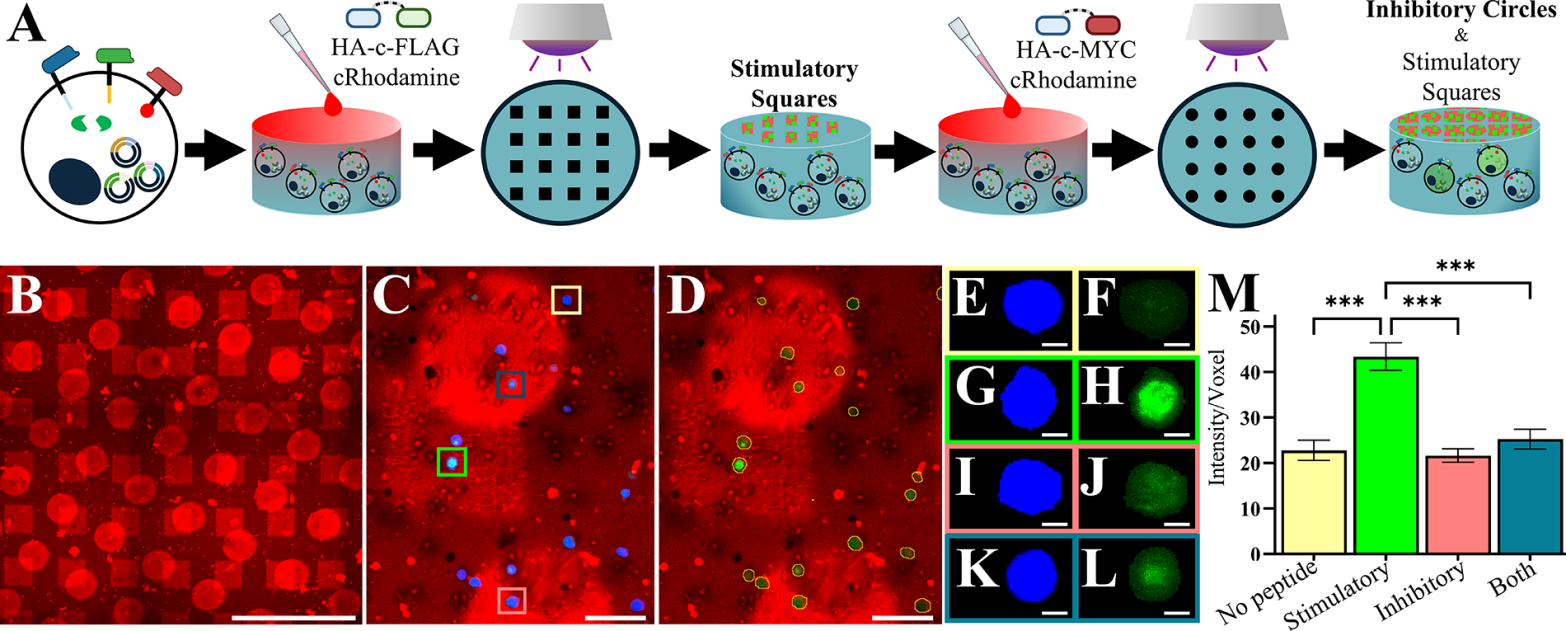
**Cells modified with Stimulatory and Inhibitory EPDA receptors spatially respond to 3D HA-c-FLAG and HA-c-MYC peptide ligands.** (A) Schematic of transfected cell encapsulation followed by inhibitory and stimulatory peptide-ligand photopatterning workflow. First, cell-laden hydrogels are soaked in a solution of photoinitiator, 50 μM HA-c-FLAG stimulatory peptide and 100 μM thiolated rhodamine peptides (cRhodamine), and peptides are patterned using a repeating square (230 μm side length) photomask. Next, unbound peptide is washed away, and hydrogels are soaked in a solution of photoinitiator, 50 μM HA-c-MYC inhibitory peptide and 100 μM cRhodamine, and peptides are patterned using a repeating circle (225 μm diameter) photomask. Hydrogels are then incubated for 4 h and imaged to evaluate platform activation (green fluorescence) of transfected cells (blue fluorescence) in and out of inhibitory circle and stimulatory square peptide treated areas. (B) Macroscopic image of rhodamine-labeled inhibitory circles and stimulatory squares. (C) Gel subsection showing rhodamine squares (red), transfected cells (blue), and activated cells (green). (D) Gel subsection with blue channel removed and transfected cell bodies demarcated as thin yellow lines. Zoomed in subsection of blue channel (left column) showing transfected cell area green channel showing platform activation (right column) within the (E & F) no peptide (yellow square), (G & H) HA-c-FLAG peptide treated area (green square), (I & J) HA-c-MYC (pink square), or (K & L) overlapping area of both peptides (blue square). Single cell analysis results of the green fluorescence intensity per voxel found within transfected cell volume outside (red) and outside (green) of stimulatory HA-c-FLAG peptide treated areas. Magnification 20x, scale bars: (B) 1 mm, (C–D) 100 μm, (E-L) 10 μm. (M) Single cell analysis results of the mean green fluorescence of transfected cells within no peptide (yellow, *n* = 50), stimulatory square (green, *n* = 50), inhibitory circle (pink, *n* = 50), and overlapping stimulatory and inhibitory (blue checkered pattern, *n* = 25) regions. Error bars: SEM, *p* < 0.001 (***).

## 3 Conclusions

The objective of this study was to develop a synthetic receptor that endows cells with the ability to sense and respond to biomaterial-laden cues. To achieve this goal, we designed Stimulatory and Inhibitory EPDA receptors that enable cells to respond to hydrogel-bound ligand peptides. Peptide ligands were designed using HA, FLAG, and MYC epitopes, and to identify ligand–receptor pairs, over 2,000 monobodies were built *in silico* using a novel PETEI algorithm. Monobodies found to have the highest theoretical peptide ligand affinity were tested experimentally, resulting in the identification of three peptide-monobody (HA-HA3, FLAG–FLAG5, MYC-MYC7) pairs. Extracellular linkers were then optimized, to develop final designs for receptors which included an intracellular zipper (H3ZA), kinase (F5KB), and phosphatase (M7PB) domain. This design-build-test-learn cycle culminated in the development of Stimulatory and Inhibitory EPDA receptor pairs that dimerize in the presence of HA-c-FLAG and HA-c-MYC peptides, respectively. Intracellularly, Stimulatory EPDA receptors phosphorylate a substrate that merges two fluorescent protein halves, whereas Inhibitory EPDA receptors revert split fluorescent proteins back to their unmerged, inactive state via substrate dephosphorylation. To demonstrate that EPDA receptors are responsive to the spatiotemporal presentation of stimulatory and inhibitory peptide ligands, transfected cells were encapsulated in HyaNor hydrogels with stimulatory and inhibitory peptide domains, and intracellular fluorescence varied based on cell location within peptide-patterned hydrogels. Here we show how this platform can be used to induce split fluorescent reconstitution in response to peptide ligands. Future work includes modifying the intracellular components of Stimulatory and Inhibitory EPDA receptors to allow for material-guided transcriptional control. This will be accomplished by replacing the split fluorescent molecules with either split dCas9 (catalytically dead Cas 9)^65–68^ halves or by attaching a synthetic DNA binding domain (e.g., zinc fingers^76^) to the tyrosine motifs and a transcriptional effector protein (e.g., VP16 ^77^) to the SH2 domain. Under this proposed scheme, EPDA receptor activation will result in the DNA binder and effector coming together, creating a functional synthetic transcription factor that upregulates transcription of a gene of interest. This will expand our platform’s therapeutic applicability by allowing for the selective expression of genes useful for regenerative medicine and immunotherapies. The EPDA receptors developed in this study also provide a versatile tool for materials scientists and synthetic biologists to investigate extracellular matrix-cell interactions and facilitate the unification of both fields.

## 4 Methods

### 4.1 Peptide Design and Synthesis

All peptides were synthesized in house via solid-phase peptide synthesis using a Liberty Blue solid-state peptide synthesizer (CEM). These include the HA-HA, FLAG–FLAG and MYC-MYC duplicated peptides, the HA-c-FLAG stimulatory and HA-c-MYC inhibitory peptides, thiolated RGD, thiolated rhodamine, and dithiolated cross-linker peptides. Immediately after synthesis peptides were cleaved from the resin (ProTide Rink, CEM) using a solution of 92.5% trifluoroacetic acid, 2.5% deionized water, 2.5% triisopropylsilane, and 2.5% 2,2-(Ethylenedioxy)­diethanethiol for 3 h. The peptides were precipitated three times in cold diethyl ether and lyophilized. Peptide synthesis was confirmed using MALDI-TOF (matrix-assisted laser desorption/ionization-time-of-flight) spectroscopy (Supplemental Figures S1, S2, S16).

### 4.2 PETEI Database Creation

PETEI was used to design monobody binding proteins for HA, FLAG, and MYC epitopes via two main steps: database generation and binder design (Supplemental Figure S21). These steps were inspired by two prior methods for computationally designing antibodies: OptCDR^45^ and OptMAVEn.^47^ OptCDR designed initial antibody structures using a database of backbone-only structures for the binding loops of the antibodies and then filled in their AAs based on energy calculations and statistical rules derived from known antibody sequences. In contrast, OptMAVEn designed initial antibody structures using an all-atom database of pieces for the entire variable domains of the antibodies. PETEI strikes a balance between these prior methods and creates an all-atom database only for the loop structures.

PETEI’s database generation step began with selecting the scaffold protein, which was chain A of the 10th type III fibronectin domain from PDB^64^ file 1TTG in this study. The binding loops that PETEI designs connect to this scaffold at attachment point residues and collectively they form a complete protein domain. The attachment points should be chosen based on an expert analysis of a protein’s structure and what portions of it are likely to be changeable. Once this scaffold protein was selected, all structures in the PDB were searched for loops that geometrically match the attachment points, namely Cα_i_-Cα_f_, Cβ_i_-Cβ_f_, Cα_i_-Cβ_f_, Cβ_i_-Cα_f_, N_i_-N_f_, O_i_-O_f_, and C_i_-C_f_ distances within 0.225 Å of those in the scaffold protein, where “i’ and ‘f” refer to the initial and final attachment point residues. The threshold of 0.225 Å was identified through trial and error as a value capable of identifying many loops while still having them appear to align with the attachment points upon visual inspection. Any loop that matched the geometric criteria was retained, regardless of whether it was part of a protein–protein interaction in the PDB.

After loops are identified from the PDB, they are scanned for structural compatibility with the scaffold they attach to as well as with the loops for other attachment points. Relatively few monobody structures have been experimentally determined, the monobody domain is homologous to the variable domains of antibodies, and the binding loops of monobodies are structurally equivalent to the Complementarity Determining Regions (CDRs) of antibodies. Therefore, analysis of the CDRs in a nonredundant database of antibody-protein complexes^78^ was used to identify quantitative cutoffs for structural compatibility. The worst 10% of antibody CDRs have 16 heavy atom (i.e., non-hydrogen) steric clashes (i.e., two atoms closer than the sum of their van der Waals (vdW) radii) and an interaction energy of 237.5 kcal/mol with the scaffold of the antibody, as calculated using the electrostatics and vdW components of the CHARMM force field.^69,70^ The PETEI-identified loops are limited to no worse than these thresholds to ensure they are comparable to CDRs. Similarly, the worst 10% of CDR pairs were found to have three heavy atom steric clashes and interaction energies of 55.6 kcal/mol with one another. PETEI-designed loops are considered compatible with one another if they do not exceed these thresholds. Finally, PETEI ensures that every loop is compatible with at least one loop from each of the other attachment points.

After a database of binding loops has been identified, PETEI positions the scaffold and the database of loops to facilitate the later design steps. Specifically, the scaffold is positioned such that the centroid of the loop attachment point is at the origin and the binding loops point up in the positive *z*-direction. The binding loops are then searched for the spatial coordinates where exceptionally strong binding interactions can occur around them, the feature that inspired PETEI’s acronym.

PETEI considers a variety of protein–protein interactions, including hydrogen bonds, salt bridges, π–π stacking, and π-cation interactions. For each of these interactions, there is a geometry that is thermodynamically optimal. For example, hydrogen bonds are strongest when there is a straight line from the hydrogen bond donor through the hydrogen to the hydrogen bond acceptor, when that line points at a lone pair of electrons on the acceptor, and when the donor and acceptor are a particular distance apart that depends on the chemical groups of the bond. Table 1 lists the interactions and corresponding ideal distances that PETEI searches for, where the distances were identified either through analyses of literature data sets or directly from prior literature reports. In addition to the distances, every type of interaction requires at least one additional geometric constraint (e.g., the vector from the hydrogen bond donor through the hydrogen for a hydrogen bond, the plane of an aromatic ring for π interactions, etc.), but the distances were the key parameters that required literature review to determine. It is likely that the side chains of AAs in binding loops will be flexible and able to assume many different conformations. PETEI uses the Dunbrack rotamer library^79^ to consider alternative AA side chain positions, the positions of all possible optimal interactions around each loop in the database are calculated and stored. Note that PETEI does not allow for mutations in the proteins it designs to ensure that the structures of the designed binding loops match their experimentally observed geometries, so the optimal interaction locations are only those for the native AA at each position in a loop. Overall, a database of binding loops made by PETEI is efficient in terms of disk space and memory requirements for use in design. *O*(*N*) PDB-formatted structures, *O*(*N* ^2^) structure compatibility data, and *O*(*N*) interaction positions are required. This format allows PETEI to complete design calculations using only a single processor rather than requiring more advanced computing resources.

**Table 1:** Interactions and Ideal Distances in PETEI Search

**interaction**	**distance (Å)**
a hydrogen bond between an N–H (donor) and O=C (acceptor)^78^	2.89
a hydrogen bond between an N–H (donor) and a HO-C (acceptor)^78^	3.00
a hydrogen bond between an O–H (donor) and a O=C (acceptor)^78^	2.79
a hydrogen bond between an O–H (donor) and a HO-C (acceptor)^78^	2.86
the distances from C to C in an ARG to ASP/GLU salt bridge^78^	3.96
the distance from N to C in a LYS to ASP/GLU bridge^78^	3.18
the distance from the central C in ARG to the center of an aromatic ring in an ARG-π interaction^80^	3.75
the distance from the N in LYS to the center of an aromatic ring in a LYS−π interaction^80^	4.20
the vertical displacements from center of ring to center of ring in a parallel displaced π–π stacking interaction^81^	3.75
the horizontal displacements from center of ring to center of ring in a parallel displaced π–π stacking interaction^65^	1.50
the distance from center of ring to center of ring in a T-shaped π–π interaction^82^	5.00
the distance from the center of histidine ring to the center of benzene ring in a parallel histidine-π interaction^80^	3.50
the distance from the center of histidine ring to the center of benzene ring in a T-shaped histidine-π interaction^80^	4.25

### 4.3 Binder Design with PETEI

The second main step of PETEI is designing binding proteins that are predicted to have exceptionally good interactions with no obvious detrimental features. Just as the generation of the database of binding loops began with the user specifying the scaffold protein the loops would attach to, binder design begins with the user specifying the structure of the target protein and identifying the specific AAs that PETEI should target (i.e., the epitope). The target protein is then oriented so that the epitope is centered above the origin in the *x*–*y* plane and points in the negative *z*-direction (i.e., toward the database of binding loops).

PETEI searches for positions of the target protein and monobody loop combinations that have enough strong interactions. The target protein has six degrees of freedom (i.e., three rotational and three translational). PETEI does not consider rotations around the x- and *y*-axes, as large rotations around these axes would point the target epitope away from the binding pocket of the designed protein. The remaining rotational degree of freedom is addressed in one-degree increments around the *z*-axis while the translational degrees of freedom are addressed simultaneously. For each rotational increment, the spatial positions where thermodynamically optimal interactions can occur around the epitope are calculated. PETEI then searches for translations of the target protein that match a user-defined minimum number (e.g., four) of complementary interactions (e.g., hydrogen bond donors with acceptors) within a tolerance of 0.33 Å. The tolerance was identified through trial and error as an appropriate balance of finding many combinations of possible interactions while remaining close to the thermodynamic optimum positions. The resulting selected interactions are not thermodynamically optimal but should be exceptionally good versions of the interactions because they are close to that optimum.

Identified monobodies with enough exceptional interactions are subsequently screened to eliminate designs with any of three types of detrimental features: steric clashes, charge–charge clashes, and excess positively charged AAs. Monobody designs with two or more steric clashes involving backbone atoms or atoms in the side chains of the residues forming the exceptional interactions are removed from consideration. For this purpose, a steric clash is defined as two heavy atoms being within 80% of the sum of their vdW radii. Note that PETEI does allow steric clashes among side chains that are not forming the targeted exceptional interactions under the assumption that those side chains would repack to eliminate the clashes. PETEI eliminates designs with any charge–charge clashes, where such clashes are defined as two atoms with the same charge (i.e., positively charged nitrogen atoms of lysine and arginine and the negatively charged oxygen atoms of aspartate and glutamate) within 4.2 Å of one another. PETEI assumes binding is occurring at physiological pH and therefore does not consider histidine as a possible charged AA. Finally, PETEI does not consider monobodies with more than three positively charged residues in the binding loops. A preliminary version of PETEI that did not include this constraint was prone to generating designs with large numbers of positively charged residues that appeared qualitatively dissimilar to antibody-protein complexes. While it is interesting to hypothesize that those proteins are still experimentally viable binders, the current version of PETEI aims to generate designs with binding modes that are qualitatively similar to natural binding interactions.

Successful PETEI designs are considered those with enough targeted exceptional interactions and no detrimental features. If a successful design does not have a selection for every binding loop, the database is searched for components that are compatible with the selected loops and do not introduce any detrimental features. PETEI also does not retain designs with greater than 60% similarity at the level of loop ID with previously identified successful designs. Here, this constraint means that each design has at most one loop that is the same as any other design.

### 4.4 Computational Evaluation of Designs

PETEI generated nearly 2,000 monobody designs, which were more than could be experimentally evaluated in this study. To determine which designs to test, further computational analysis was conducted. All complexes were first minimized using the CHARMM force field^83,84^ with fixed backbone atoms to repack clashing side chains. The complexes were subsequently all-atom minimized using the Rosetta force field and the Rosetta Interface Analyzer was used to calculate predicted binding energies and buried surface areas. CHARMM was used first because it was found to be able to successfully resolve side chain steric clashes that PETEI introduces much more frequently than Rosetta. Once CHARMM had resolved those clashes, Rosetta was used to minimize the entire complexes and calculate the properties because it is the most used protein force field. In addition to the PETEI-generated designs, the nonredundant antibody-protein database was prepared using the same protocol to provide a comparison for the calculated values.

### 4.5 Plasmid Designs

All plasmids are provided as GenBank files in Supplemental Zip 2, and Supplemental Workbook 1 includes the AA sequences and promoters for each plasmid. The PR, PD, CDZK, ZPC as well as the kinase, zipper, HA1–10, FLAG1–10, MYC1–10, H3ZA, F5SB, and M7PB receptor components were constructed using molecular cloning procedures and validated using Sanger sequencing or PCR (polymerase chain reaction). Empty plasmid,^76^ eBFP, and miRFP720 transfection control plasmids were gifts from Joshua Leonard. All plasmids were prepared using the Monarch Plasmid Miniprep Kit, eluted in 10 mM Tris and 0.1 mM EDTA, and stored frozen until use in transfection.

### 4.6 Transient Transfection of Mammalian Cells

HEK293 (passage P3–P7) cells are plated in 500 μL of culture media (DMEM supplemented with 10% fetal bovine serum and 1% penicillin-streptomycin) at a concentration of 140,000 cells/well in 24 well plates. These cells are allowed to settle and spread in an incubator at 37 °C, 5% CO_2_ for 10 h before transfection. Each transfection includes DNA plasmid concentrations of 75 ng of PR, 125 ng of PD, 125 ng of the zipper and kinase receptors, 75 ng of ZPC, 75 ng of a miRFP720 fluorescent protein transfection control plasmid, and an empty plasmid to reach a final DNA concentration of 700 ng, as done in previous studies.^85^ The positive control received 125 ng of CDZK in place of the receptors. This DNA was then mixed with 4 μL of 2.5 M CaCl_2_ and diH_2_O to a total volume of 50 μL. This mixture was added to 50 μL of 2x HEPES Buffer Saline (pH 7.1) dropwise and allowed to incubate at room temperature for 3 min. This mixture was then vigorously mixed to thoroughly break up particles and immediately added dropwise to the HEK293 cells. After a 15-h incubation, the media was aspirated and unless otherwise noted the ligand of rapamycin or the peptide was added at this time in 1 mL of culture media. After 24 h, the media is aspirated, the cells are detached using a solution of PBS-EDTA and assayed using a flow cytometer. Single cells were gated using forward and side scatter and only single cells which expressed the miRFP720 transfection control above the empty plasmid control were reported. All experiments recorded on the flow cytometer were performed in biological quadruplicate and each sample group produced at least 10,000 transfected cells. The mean GFP fluorescence emission levels of each sample were measured and reported in arbitrary units between the value of the background only samples (PR and PD) and the positive control (PR, PD, and CDZK).

For 3D hydrogel studies, HEK293 cells were plated at 600,000 cells/well in a 6 well plate and allowed to spread for 10 h and transiently transfected using Lipofectamine 3000 (Invitrogen). All DNA plasmids and their weights per well were kept the same as in previous experiments, with the exception that miRFP720 was replaced with eBFP so that transfected cells can be visualized using a 488 nm confocal microscope laser.^86^ DNA was added to 25 μL of Opti-MEM (Invitrogen), then mixed with a solution of 0.5 μL Lipofectamine 3000, 0.5 μL P3000 reagent in 25 μL Opti-MEM per well and allowed to incubate at room temperature for 20 min before adding the total 50 μL to each well. 16 h after lipofectamine treatment, culture media was aspirated and replaced with 500 μL of fresh culture media.

### 4.7 Split Fluorescent Reporter Qualification

To determine the effect that constitutive kinase and LZRR proximity has on fluorescence and to set a positive control for each experiment, the kinase domain was attached directly to the LZRR (Figure 1C). This constitutively dimerized LZRR and kinase positive control (CDZK) binds reversibly with the leucine zipper of the phosphorylation recipient and attaches a phosphoryl group to its tyrosine residues (Figure 1 **Di** **-ii**). Next, the SH2 domain on the phosphorylation detector will dimerize with the tyrosine residues, allowing the split GFP halves to reconstitute and fluoresce green (Figure 1 **Diii-iv**). The negative control for all experiments is the phosphorylation recipient and phosphorylation detector transfected alone with no corresponding zipper or kinase to set a baseline of split reconstitution. To reduce background dimerization in peptide receptor experiments a constitutively dimerized zipper and protein tyrosine phosphatase 1B is used (Figure 1 **E-F** **)**.^57,58^

### 4.8 Homodimer Monobody Receptor Screening and Optimization Using Duplicated Peptides

To determine which of the top 10 monobodies were successful as transmembrane receptors they were tested by matching the same monobody on the leucine zipper receptor and the kinase receptor. These cells were treated with the duplicated peptides with the epitope corresponding to these monobodies. The monobodies which experienced ligand dependent homodimerization would show an increase in their GFP fluorescence emission. Those which received the highest fold change in fluorescence would be selected for further optimization of extracellular linker lengths. For this optimization, the monobodies were kept the same (HA3, FLAG5, and MYC7) and 10, 20, and 30 AA linker lengths were evaluated. The receptors which showed the most consistent and potent increases in fluorescence were selected for future tests where they could be used as heterodimers and within materials.

### 4.9 Heterodimer Receptor Kinematics

Once the optimal monobody receptor and linkers were determined for both the kinase and zipper receptor halves, they were tested together as heterodimers. This should improve ligand dependent activity because it eliminates the potential for the occurrence of nonproductive receptor dimerization where a zipper receptor binds to itself instead of a kinase or vice versa in response to duplicated peptide ligand activation. For this testing the HA-c-FLAG peptide ligand was utilized. After ensuring that ligand–receptor pairs were effective at dimerizing both receptor halves, we evaluated ligand–receptor dynamics. First, the optimal ligand treatment time was tested by treating the cells with 100 μM of HA-c-FLAG peptide 0, 1, 2, 4, 6, 12, and 24 h before analysis. For all time points the culture media was changed 15 h after transfection and changed again to administer the ligand, keeping the total time after transfection consistent at 39 h to give the cells enough time to transcribe and express the receptors at the surface. Next, concentration (25, 50, and 100 μM of the HA-c-FLAG peptide) was optimized at the first time point which saw a significant increase in green fluorescence (1 h). Peptide concentrations of 200 μM tended to reduce cell health and are not reported.

### 4.10 HyaNor Macromer Synthesis

To form biocompatible hydrogels that can be photopatterned with peptide ligands via thiol-norbornene click chemistry reactions, hyaluronic acid macromers were modified with norbornene (HyaNor) as described previously.^26^ Briefly, sodium hyaluronate (NaHA, 2% w/v) was first converted to its tetrabutylammonium salt (HA-TBA). Carboxyl groups in HA-TBA were then modified with norbornenes via amidation with 5-norbornene-2-methylamine in an anhydrous solution of dimethyl sulfoxide (DMSO, 2% w/v) and benzotriazole-1-yl-oxy-tris­(dimethylamino)-phosphonium hexafluorophosphate (BOP) under nitrogen for 2 h at room temperature. The reaction was quenched with cold water, dialyzed (SpectraPor, 6–8 kDa molecular weight cutoff), frozen, and lyophilized. HyaNor macromer was analyzed using ^1^H NMR spectroscopy and modification was determined by integrating the norbornene peaks between 5.8 and 6.0 ppm (Supplemental Figure S3).

### 4.11 HyaNor Hydrogel Synthesis and Characterization

All hydrogels were formed using 3 wt % HyaNor, 0.05 wt % I2959, 2 mM thiolated RGD peptide (GCGYGRGDSPG), and 3 mM dithiolated cross-linker peptide (GCNSVPMSMRGGSNCG). The dithiolated cross-linker peptide used in this study is biocompatible and has been used by our group and others.^23,87^ Hydrogels containing no peptide, 100 μM HA-c-FLAG, 100 μM HA-c-MYC, or 100 μM thiolated rhodamine peptides underwent unconfined compression testing to determine the bulk elastic modulus for each group. Cell viability was tested using hydrogels containing 1 million cells/mL and 100 μM of the HA-c-FLAG peptide incubated in culture media for 48 h. After 2 days, mammalian cell LIVE/DEAD Viability/Cytotoxicity Kit (Invitrogen) was added to growth media per the manufacturer’s instructions. Hydrogels were incubated in this solution for 30 min, then replaced with growth media, and samples were imaged using confocal microscopy within 2 h. Live and dead cells were quantified by counting green and red individual cells, respectively. For 3D cell-hydrogel studies, 24 h after Lipofectamine treatment, transfected cells were detached with trypsin-EDTA (0.05 wt %, 5 min), immediately resuspended in media, and counted. Cell solutions of known count in culture media were then centrifuged (500 RCF, 5 min), cell culture media was aspirated, and cell pellets were resuspended in hydrogel solution to achieve a hydrogel cell solution concentration of 5,000,000 cells/mL. To form hydrogels, 50 μL of this solution was placed into cylindrical molds (8 mm diameter, 0.5 mm thickness) and irradiated with UV light (6 mW/cm^2^, 5 min). Hydrogels were then removed from molds, rinsed with PBS, and cultured in culture media in an incubator (37 °C, 5% CO_2_).

### 4.12 Spatial Tethering of Peptide Ligands onto Cell-Laden HyaNor Hydrogels

To evaluate 3D cell-material interactions between EPDA transfected cells and peptide ligand-functionalized hydrogels, HEK293 cells (5,000,000 cells/mL) were encapsulated in HyaNor hydrogels for 20 h prior to peptide photopatterning. For stimulatory peptide patterning, cell-laden HyaNor hydrogels were soaked in a stimulatory peptide solution (PBS, 0.05 wt % I2959, 100 μM HA-c-FLAG peptide, and 100 μM thiolated rhodamine to visualize patterns) for 10 min, a square photomask (230 μm side length) was placed on top, and hydrogels were irradiated with UV light (5 mW/cm^2^, 5 min). For the stimulatory plus inhibitory peptide patterning, cell-laden HyaNor hydrogels were first soaked in a stimulatory peptide solution (PBS, 0.05 wt % I2959, 50 μM HA-c-FLAG peptide, and 100 μM thiolated rhodamine to visualize patterns) for 10 min, a square photomask (230 μm side length) was placed on top, and hydrogels were irradiated with UV light (5 mW/cm^2^, 5 min). After washing unbound peptides, HyaNor hydrogels with stimulatory square patterns were soaked in an inhibitory peptide solution (PBS, 0.05 wt % I2959, 50 μM HA-c-MYC peptide, and 100 μM thiolated rhodamine to visualize patterns) for 10 min, a circle photomask (225 μm diameter) was placed on top, and hydrogels were irradiated with UV light (5 mW/cm^2^, 5 min). For both studies, samples were cultured in culture media for 4 h, then washed twice with PBS, fixed with 4% paraformaldehyde (30 min), washed twice with PBS, and imaged with a confocal microscope.

### 4.13 Volumetric Analysis of Peptide-Ligand Interactions with EPDA Receptors in 3D Hydrogels

For stimulatory peptide hydrogel analysis, 3D confocal image stacks (20× magnification, ∼ 100 μm depth, 0.85 μm *z*-axis step-size) were converted to 8-bit images using ImageJ software (version 1.54i). For each image, blue (eBFP cell transfection control), green (split GFP protein), and red (stimulatory peptide patterns) channels were acquired. The eBFP signal was binarized using Otsu-based thresholding to determine the eBFP expressing cell volume for each stack. This was followed by a dilation to create a 3D cell mask, which was superimposed on the green channel for each stack. The 3D object counter was used to determine individual cell volumes (μm^3^) and the number of voxels using the binary blue channel and the integrated pixel density intensity within each cell volume of the masked green channel. Green fluorescence intensity was calculated by dividing the cell integrated density by the number of cell voxels. Analyzed cells were split into those found within stimulatory peptide (red square), inhibitory (circles) regions, in addition to cells exposed to stimulatory and inhibitory peptides as well as peptide-free regions.

### 4.14 Statistics

The binding energies per buried surface area for each receptor epitope pair were compared to the database of antibodies using two-tailed *t* tests assuming unequal variances (α = 0.05). All soluble peptide experiments were done in biological quadruplicate and multiple unpaired *t* tests were performed between the no treatment and treatment groups (α = 0.05). The single cell analysis for the stimulatory peptide experiment included a sample size of *n* > 100, and a two tailed *t* test was used to compare the fluorescence values for cells found within versus outside of the peptide treated areas (α = 0.05). The analysis for the orthogonal peptide experiment was performed using an ordinary one-way ANOVA with a posthoc Tukey *t* test to compare differences between groups (α = 0.05). Bar graphs represent the mean, and the error bars represent ± the standard error of the mean. Differences among groups are stated as * *p* < 0.05, ** *p* < 0.01, and *** *p* < 0.001.

## Supplementary Material











## Data Availability

The code and resources necessary to run PETEI to design 10th Type III Fibronectin Domains are available under the CC BY-NC 4.0 License at https://github.com/rjp0029/PETEI. This includes the database of binding loops and the scaffold used for the generation of the designs in this study.

## Notes

Supporting Information: Figures S1 to S21 and Table S1 (PDF)
Supplemental Workbook 1: Plasmid AA sequences and promoters, and database of PETEI binding loops (XLSX)
Supplemental Zip 1: PDB files of predicted monobody–epitope complexes (ZIP)
Supplemental Zip 2: GenBank files used for creating plasmids used in this study (ZIP)
Supplemental Video 1: Multiple angles of stimulatory EPDA cells found within and surrounding the stimulatory square region (MP4)
