# Supplementary material for: Extracellular Peptide-Ligand Dimerization Actuator Receptor Design for Reversible and Spatially Dosed 3D Cell-Material Communication
Source: ACS Synth Biol. 2024 Dec 20;14(7):2494–513. doi: 10.1021/acssynbio.4c00482 (PMC12281622; doi:10.1021/acssynbio.4c00482)
Supplement: Supplementary file 1 [file sb4c00482_si_001.pdf]

Supplemental Information

Extracellular Peptide-Ligand Dimerization Actuator (EPDA) Receptor Design for Reversible and Spatially Dosed 3D Cell-Material Communication

Matthias Recktenwald[1,*], Ritankar Bhattacharya[2,*], Mohammed Mehdi Benmassaoud[1], James MacAulay[1], Varun M. Chauhan[2], Leah Davis[1], Evan Hutt[1], Peter A. Galie[1], Mary M. Staehle[1], Nichole M. Daringer[1], Robert J. Pantazes[2], Sebastián L. Vega[1,3,#]

1 Department of Biomedical Engineering, Rowan University, 201 Mullica Hill Rd, Glassboro, NJ, 08028, USA

2 Department of Chemical Engineering, Auburn University, Auburn, AL, 36849, USA

3 Department of Orthopaedic Surgery, Cooper Medical School of Rowan University, Camden, NJ, 08103, USA

* These authors contributed equally to this work

# Correspondence: vegas@rowan.edu, 856-256-5522

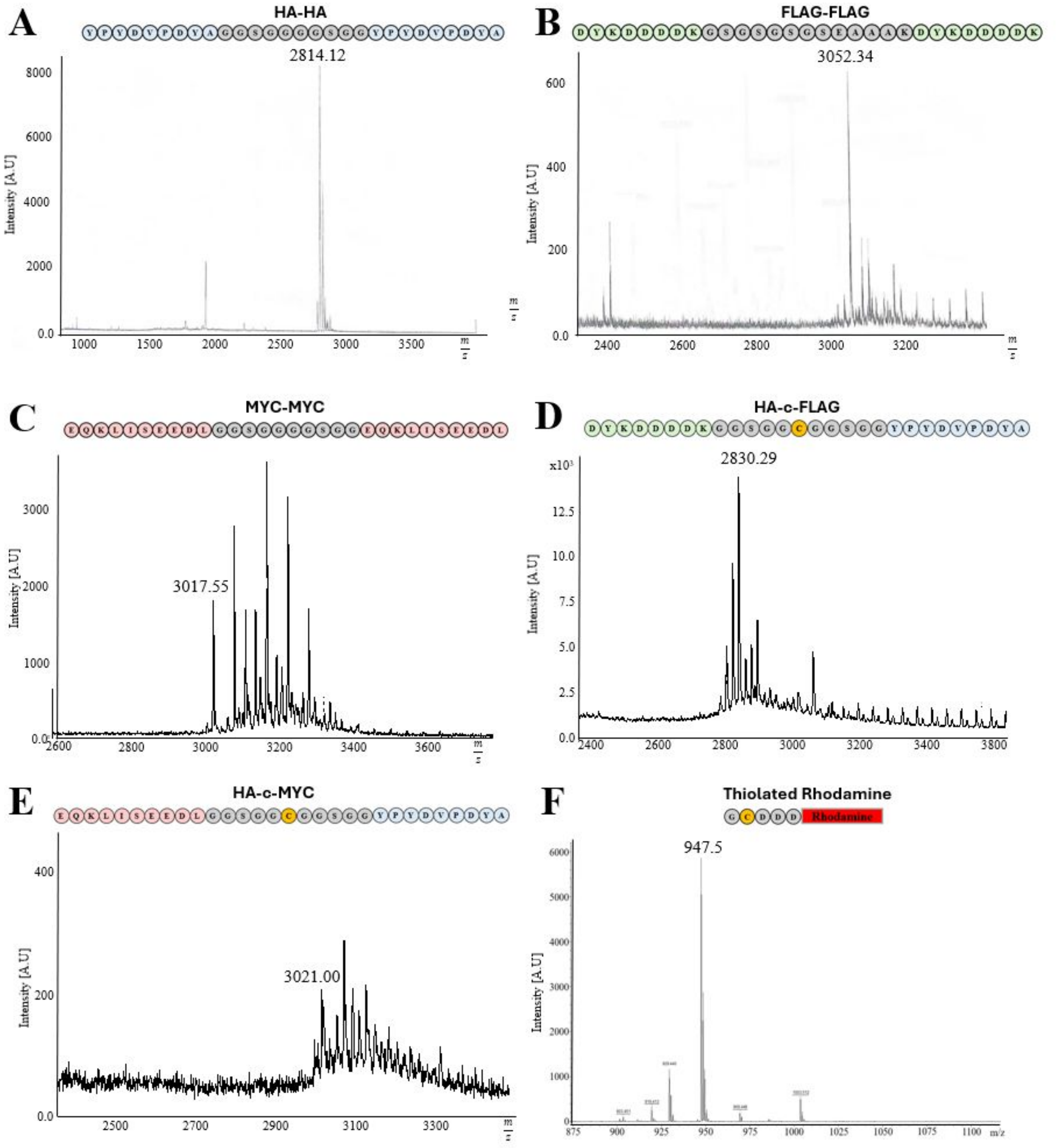

Supplemental Figure S1: MALDI-TOF Spectrometry of Synthesized Peptides. (A) HA-HA MALDI-TOF mass spectrometry results. Most intense peak: 2814.12 *m/z*; expected peak: 2815.90 *m/z*. **(B)** FLAG-FLAG MALDI-TOF mass spectrometry results. Most intense peak: 3052.34 *m/z*; expected peak: 3054.0*m/z*. **(C)** MYC-MYC MALDI-TOF mass spectrometry results. First high intensity peak: 3017.55 *m/z*; expected peak: 3018.19 *m/z*. **(D)** Thiolated HA-c-FLAG MALDI-TOF mass spectrometry results. Most intense peak: 2830.29 *m/z*; expected peak: 2829.85 *m/z*. **(E)** Thiolated HA-c-MYC MALDI-TOF mass spectrometry results. First high intensity peak: 3021.00 *m/z*; expected peak: 3020.19 *m/z*. **(F)** Thiolated Rhodamine MALDI-TOF mass spectrometry results. Most intense peak: 947.5 *m/z*; expected peak: 947.5 *m/z*.

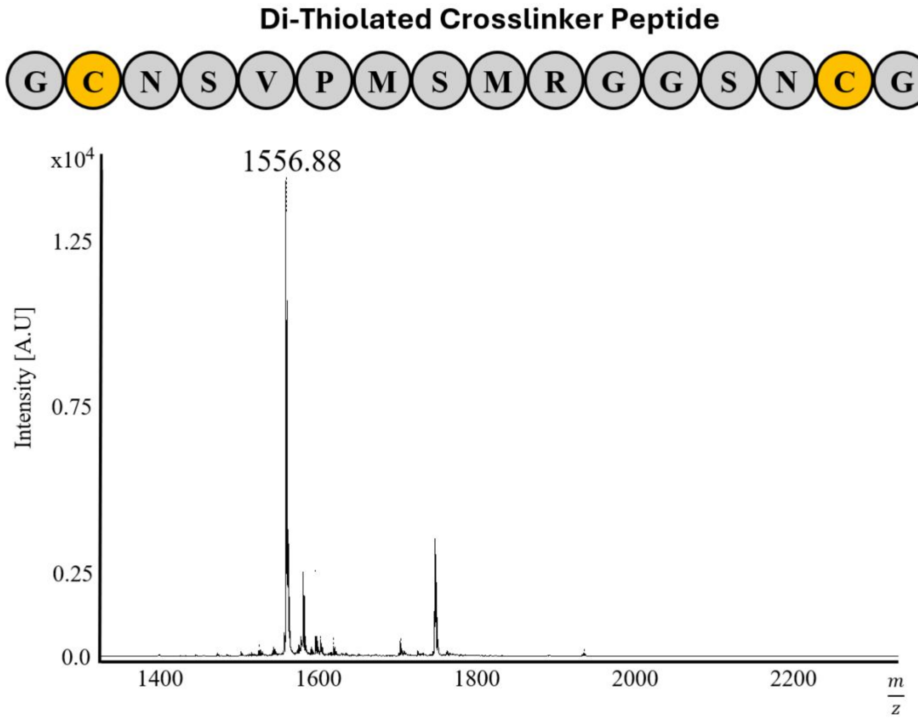

Supplemental Figure S2: MALDI-TOF Spectrometry of Di-Thiolated Crosslinker Peptides. Di-thiolated crosslinker peptide used to form HyaNor hydrogels MALDI-TOF mass spectrometry results. Most intense peak: 1556.88 *m/z*; expected peak: 1557 *m/z*.

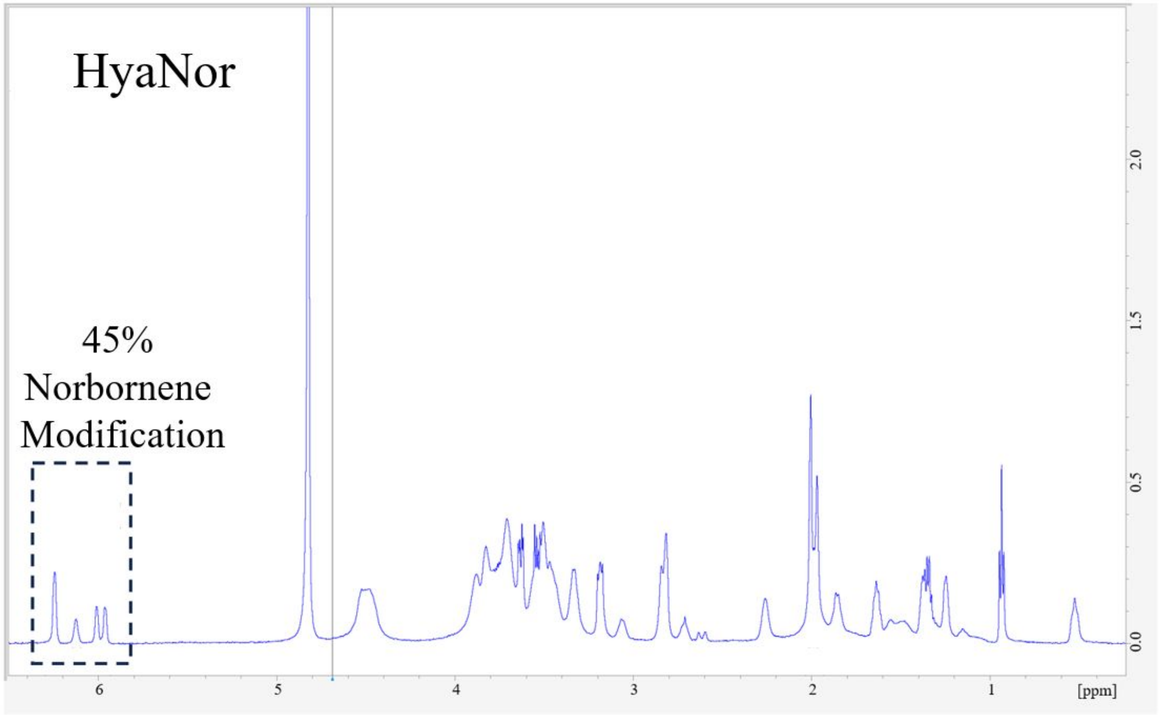

Supplemental Figure S3: HyaNor NMR Spectra. ^{1}H NMR spectra of HyaNor macromer shows that ~45% of Hya repeat units are functionalized with norbornene.

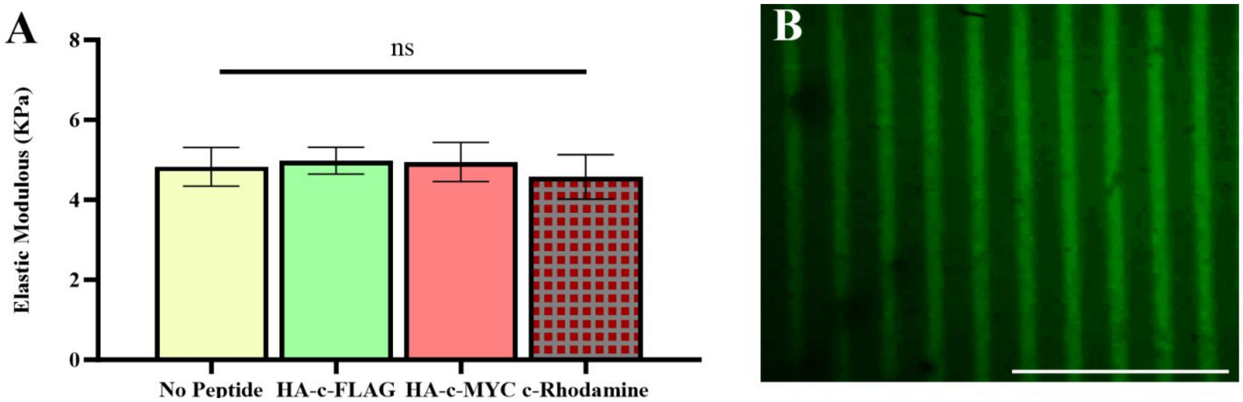

Supplemental Figure S4: HyaNor Hydrogel Peptide Tethering Studies. (A) Elastic moduli (kPa) for HyaNor hydrogels formed with no peptide (4.8 kPa) or with 100 µM HA-c-FLAG (5.0 kPa), 100 µM HA-c-MYC (5.0 kPa), or 100 µM thiolated rhodamine (4.6 kPa) peptides. There is no statistical difference between the groups, demonstrating that the inclusion of peptides does not affect mechanics. **(B)** HyaNor hydrogels underwent a secondary reaction with mono-thiolated carboxyfluorescein-HA-c-FLAG peptides (100 µM) using a photomask (vertical stripes, 50 µm width, 60 µm spacing), photoinitiator (I2959, 0.05 wt%) and UV light (5 mW/cm^2, 5 min). This resulted in a HyaNor hydrogel photopatterned with fluorescent HA-c-FLAG peptide-ligands in regions exposed to light. n = 8 hydrogels/group, ns denotes no statistical significance. Scale bar: B = 500 µm.

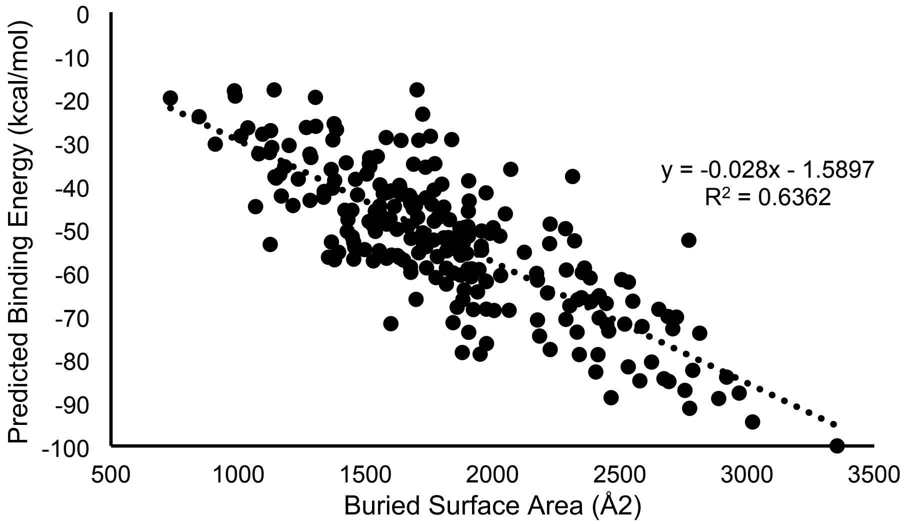

Supplemental Figure 5: Predicted Binding Energy versus Buried Surface Area for a Non-redundant Database of Antibody-Protein Complexes. The data demonstrates that predicted binding energies strengthen (i.e., become more negative) as buried surface area increases, which is a phenomena that is know to not occur in experimental tests. This correlation caused us to rank order designs on the basis of their binding energies per buried surface area for experimental testing.

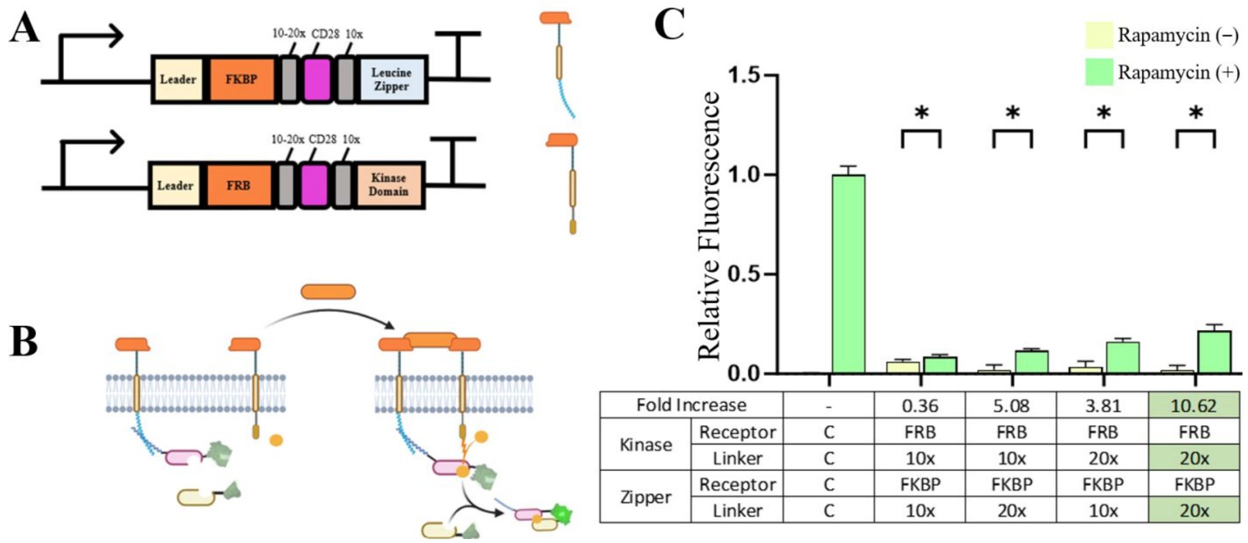

Fold Increase		-	0.36	5.08	3.81	10.62
Kinase	Receptor	C	FRB	FRB	FRB	FRB
	Linker	C	10x	10x	20x	20x
Zipper	Receptor	C	FKBP	FKBP	FKBP	FKBP
	Linker	C	10x	20x	10x	20x

Supplemental Figure S6: Transmembrane Receptors that Dimerize in the Presence of Rapamycin Ligands. (A) Rapamycin-inducible receptor design. (Top) extracellular domain consists of a FKBP12 ligand binding protein (FKBP) and a 10x or 20x linker, with an intracellular leucine zipper. (Bottom) extracellular domain consists of a rapamycin binding protein (FRB) and a 10x or 20x linker, with an intracellular kinase. **(B)** Schematic of rapamycin induced dimerization and split fluorescent reconstitution. **(C)** Flow cytometry data of rapamycin (100 nM) induced platform activation with different extracellular linker lengths. The 20 AA extracellular linker zipper and 20 AA kinase receptor pair performed best with a 1,062%-fold increase upon rapamycin activation. Error bars represent standard error around the mean (s.e.m.); * $p < 0.05$; n=4 for each group, with at least 10,000 cells assayed per Rapamycin (-) and Rapamycin (+) treatment via flow cytometry.

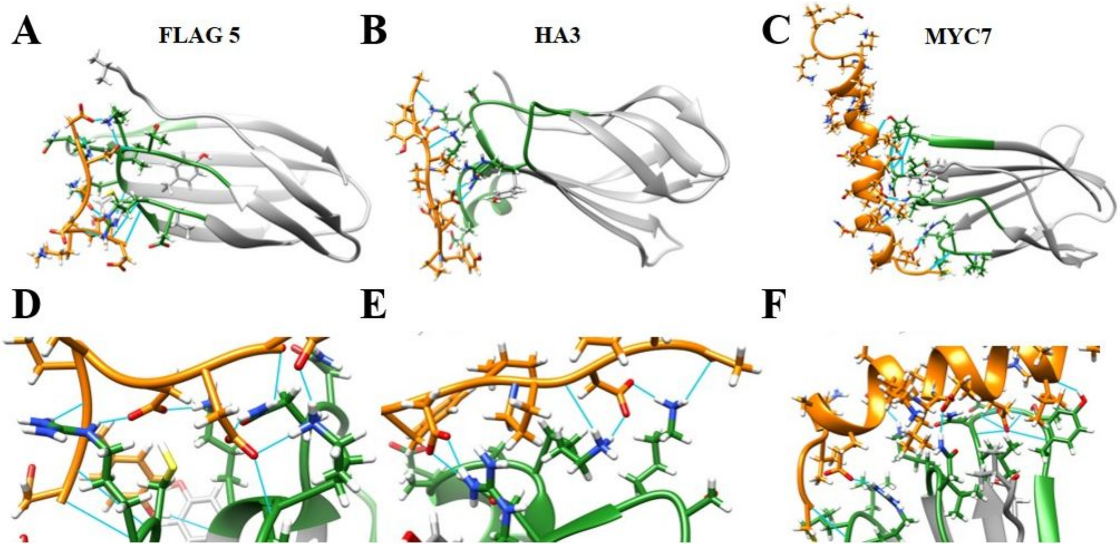

Supplemental Figure S7: Structures of the FLAG5, HA3, and MYC7 Monobody-Peptide Complexes. The scaffolds of the monobodies are shown in gray, binding loops in green, and target peptides in orange. All amino acid side chains in the peptides are shown, as are those of monobody residues within 5 Å of the peptide. Oxygen atoms are shown in red, nitrogen atoms in blue, and hydrogen bonds as cyan lines. Shown are **(A-C)** overviews of the complexes as a whole and **(D-E)** close-up views of the monobody receptor-peptide ligand interfaces.

A

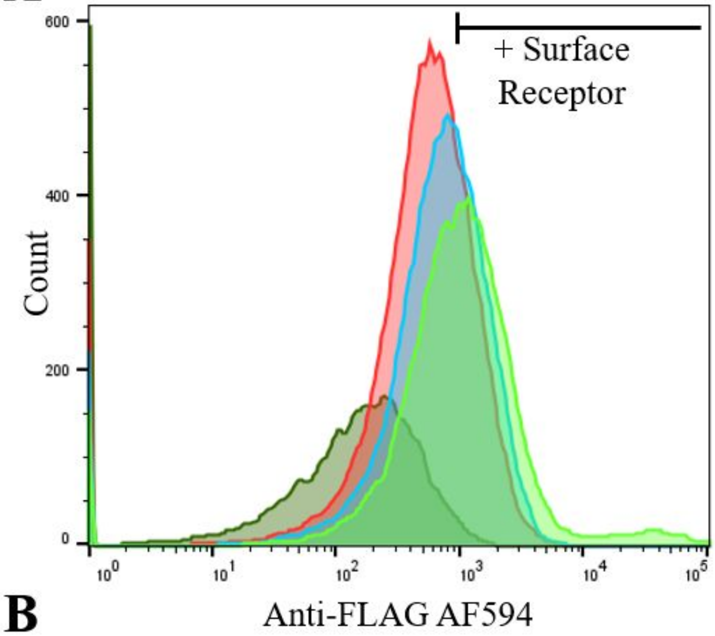

B

Receptor	Total Cells	Cells expressing surface tag	% cells
No Receptor Control	19847	397	2
H3ZA	21810	9378	43
F5KB	25355	14452	57
M7PB	14599	4380	30

Supplemental Figure S8: Extracellular Surface Tagged Receptor Staining. The flag tag sequence was cloned onto the monobody receptors. Cells were transfected using Lipofectamine 3000 with 100 ng of each tagged receptor. 40 hours after transfection cells were treated with anti-FLAG AF594 antibody, rinsed 2x and ran on the flow cytometer. (**A**) All three of these receptors showed an increase in the percentage of cells expressing the FLAG tag on the surface. **(B)** Background receptor expression is marked where minimal (2%) control sample cells, transfected with empty plasmid, exhibit AF594 fluorescence. 43% of HEK293 cells with a HA3 monobody, a 10 AA extracellular linker, and intracellular leucine zipper expressed the H3ZA receptor, as evidenced by increase in AF594 fluorescence above background. 57% of HEK293 cells with a FLAG5 monobody, a 20 AA extracellular linker, and intracellular kinase expressed the F5KB receptor, as evidenced by increase in AF594 fluorescence above background. 30% of HEK293 cells with a MYC7 monobody, a 20 AA extracellular linker, and intracellular phosphatase expressed the M7PB receptor, as evidenced by increase in AF594 fluorescence above background.

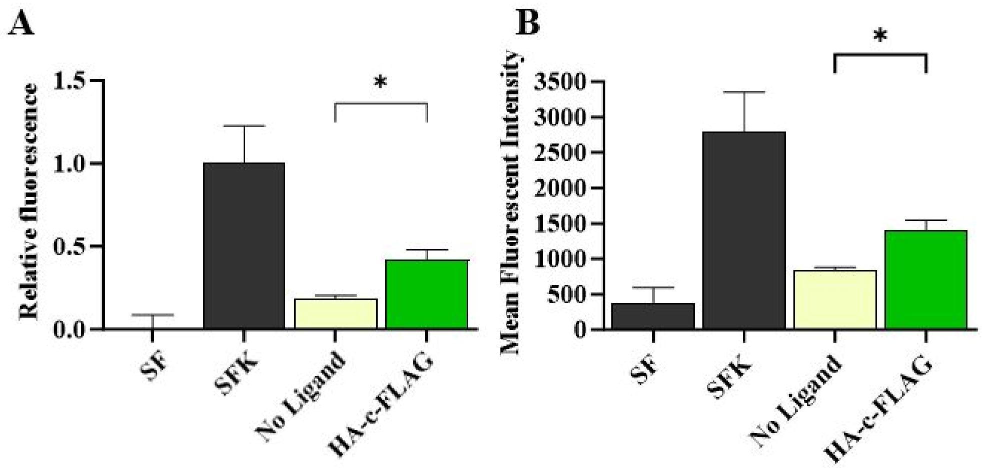

Supplemental Figure S9: Representative MFI Value for Stimulatory EPDA Receptor Platform. (A) Relative fluorescence value as shown in Figure 6 where the no ligand and HA-c-FLAG treated samples are represented as values between the negative (SF) and positive (SFK) controls. (B) Mean fluorescent intensity values of both controls and the no ligand and HA-c-FLAG treated samples from the same experiment. Error bars represent standard error around the mean (s.e.m.); * p < 0.05; n=4 for each group, with at least 10,000 cells assayed per treatment group via flow cytometry.

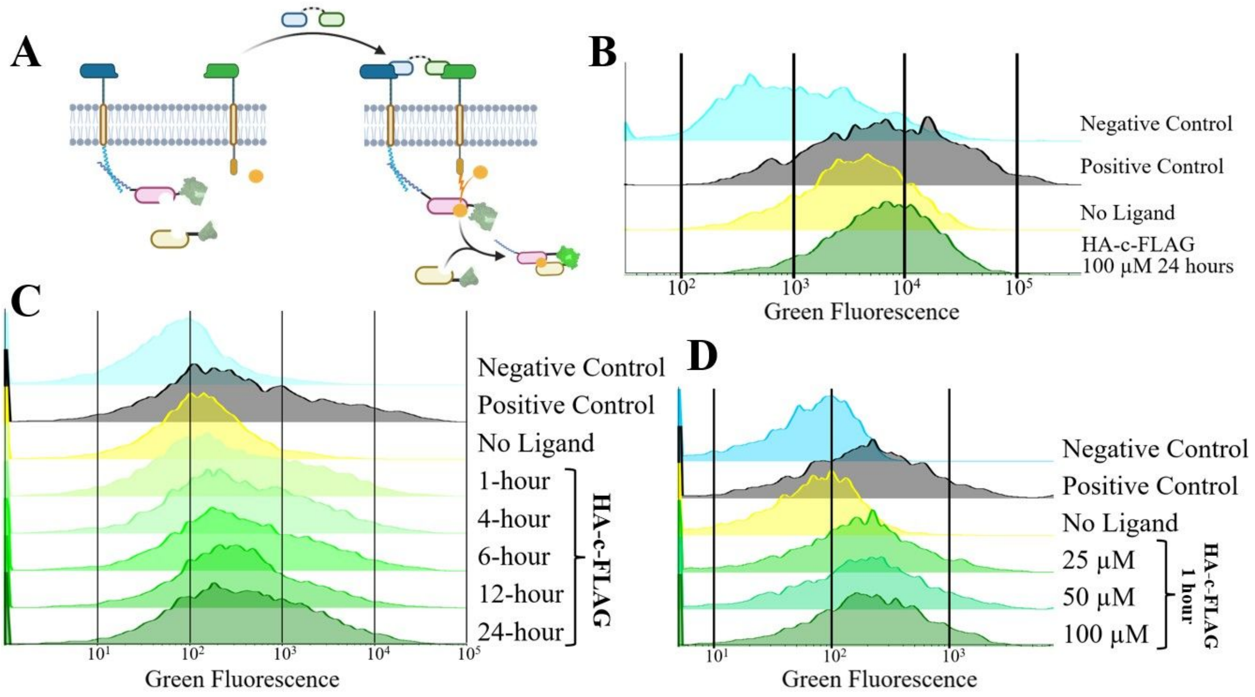

Supplemental Figure S10: Histograms From HA-c-FLAG Receptor Flow Cytometry Data: **(A)** Schematic of HA-c-FLAG peptide heterodimer activating intracellular GFP merging via HA3 and FLAG5 interactions with corresponding zipper and kinase monobodies in a Stimulatory EPDA receptor. Representative histograms of the green fluorescence from control groups and HZ3A and F5KB receptor groups treated with no peptide or the HA-c-FLAG peptide for **(B)** 24 hours and at varying **(C)** treatment times and **(D)** concentrations.

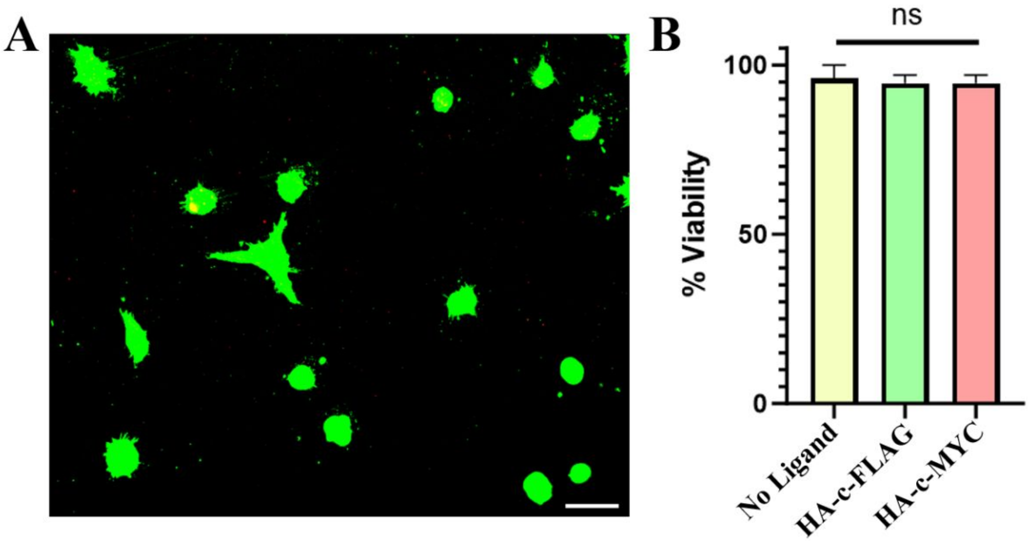

Supplemental Figure S11: Viability Assessment of Peptide-Ligand Treated Cells. (A) Live (green) and dead (red) stained HEK293 cells after 36 hours of treatment with 100 µM of peptide ligand in solution. Cells were seeded at 5,000 cells/cm^2. Images were taken using confocal microscopy and at least 25 cells were counted per sample with three samples per group. Scale bar: 100 µm, magnification: 20x **(B)** Viability was calculated by dividing the number of green cells by the total number of cells (red and green cells) per sample. Viability was 96% for no ligand treated cells, 95% for HA-c-FLAG, and 95% for HA-c-MYC treated cells. Error bars represent standard deviation; ns denotes no statistical significance across groups.

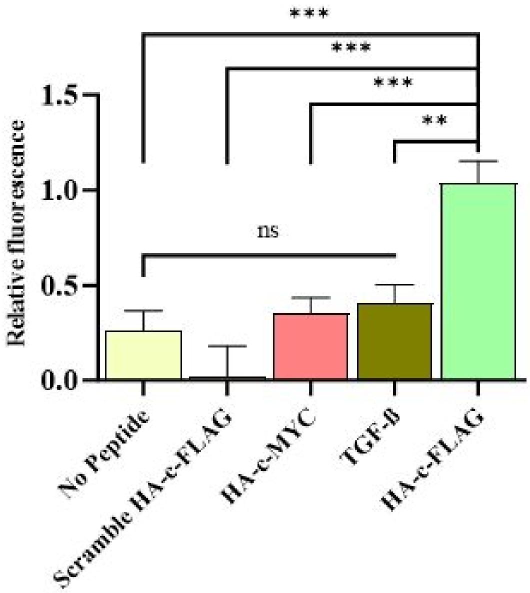

Supplemental Figure S12: Stimultory Peptide Receptor Selectivity. While the HA-c-FlAG peptide shows significant platform activation above all groups including the no peptide treated samples there is a lack of significant platform activation above the no peptide treated group for a scrambled HA-c-FLAG peptide, the inhibitory HA-c-MYC peptide or TGF-β, a full legnth cytokine dimer. Error bars represent standard error around the mean (s.e.m.); ** $p < 0.01$, *** $p < 0.001$; n=4 for each group, with at least 10,000 cells assayed per treatment group via flow cytometry.

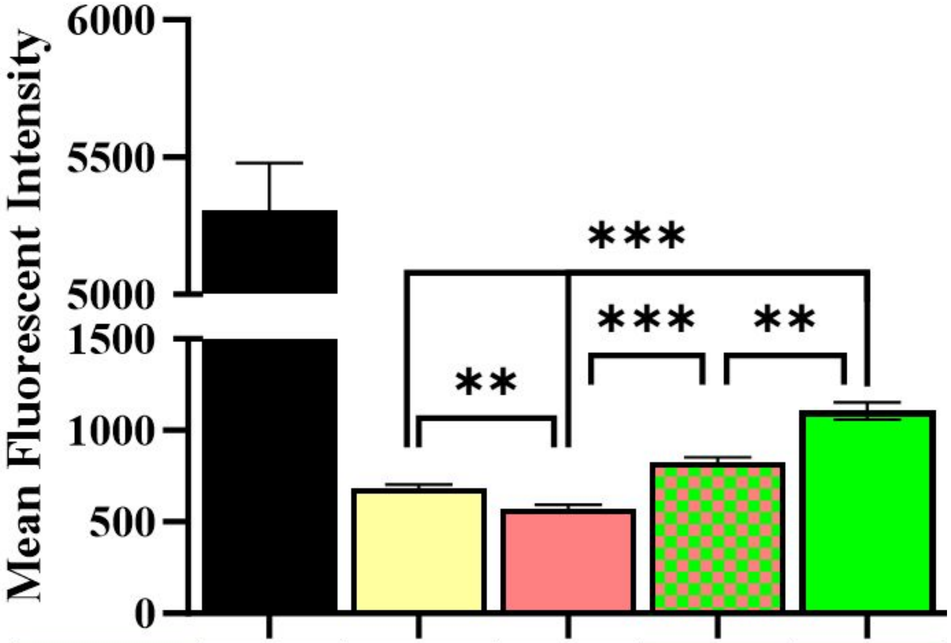

HA-c-FLAG (µM)	-	0	0	50	50
HA-c-MYC (µM)	-	0	50.0	50.0	0
Zipper	Control	HA3	HA3	HA3	HA3
Kinase	Control	FLAG5	FLAG5	FLAG5	FLAG5
Phosphatase	-	MYC7	MYC7	MYC7	MYC7

Supplemental Figure S13: Representative MFI Value for Reversible EPDA Receptor Platform. Mean fluorescent intensity plot shows low activation in the absence of peptide or in the presence of 50 µM HA-c-MYC peptide. In contrast, in the presence of stimulatory HA-c-FLAG peptide (50 µM) there is a 61% increase in fluorescence over the no peptide ligand group and a 93% increase in MFI over the equimolar treatment. Error bars represent standard error around the mean (s.e.m.); ** $p < 0.01$, *** $p < 0.001$; n=4 for each group, with at least 10,000 cells assayed per treatment group via flow cytometry.

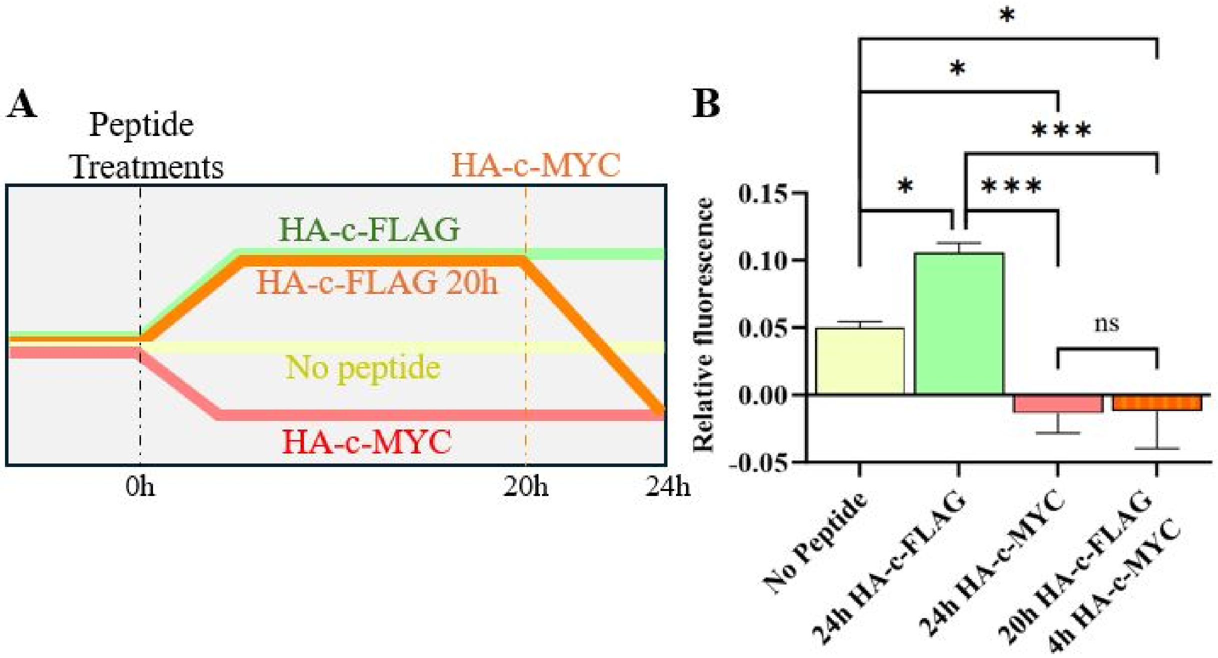

Supplemental Figure S14: Temporal EPDA Inactivation with Inhibitory Peptides Post-Stimulatory Peptide Activation. (A) Representative schematic of experimental design with treatment conditions. The yellow line represents the no peptide treatment group. The green line represents the stimulatory peptide treatment group which received 100 µM of HA-c-FLAG peptide for 24 hours. The red line represents the inhibitory peptide treatment group which received 100 µM of HA-c-MYC peptide for 24 hours. The orange line represents the stimulatory/inhibitory peptide treatment group which received 100 µM of stimulatory HA-c-FLAG peptide for 20 hours followed by 100 µM of inhibitory HA-c-MYC peptide for 4 hours. **(B)** Experimental results show a ~100% fold increase in platform activation in the stimulatory peptide treatment group, and relative fluorescence levels slightly below background for both the inhibitory and stimulatory/inhibitory peptide (HA-c-FLAG for 20 hours followed by HA-c-MYC for 4 hours) treatment groups. Error bars represent standard error around the mean (s.e.m.); * $p < 0.05$, *** $p < 0.001$; n=4 for each group, with at least 10,000 cells assayed per treatment group via flow cytometry.

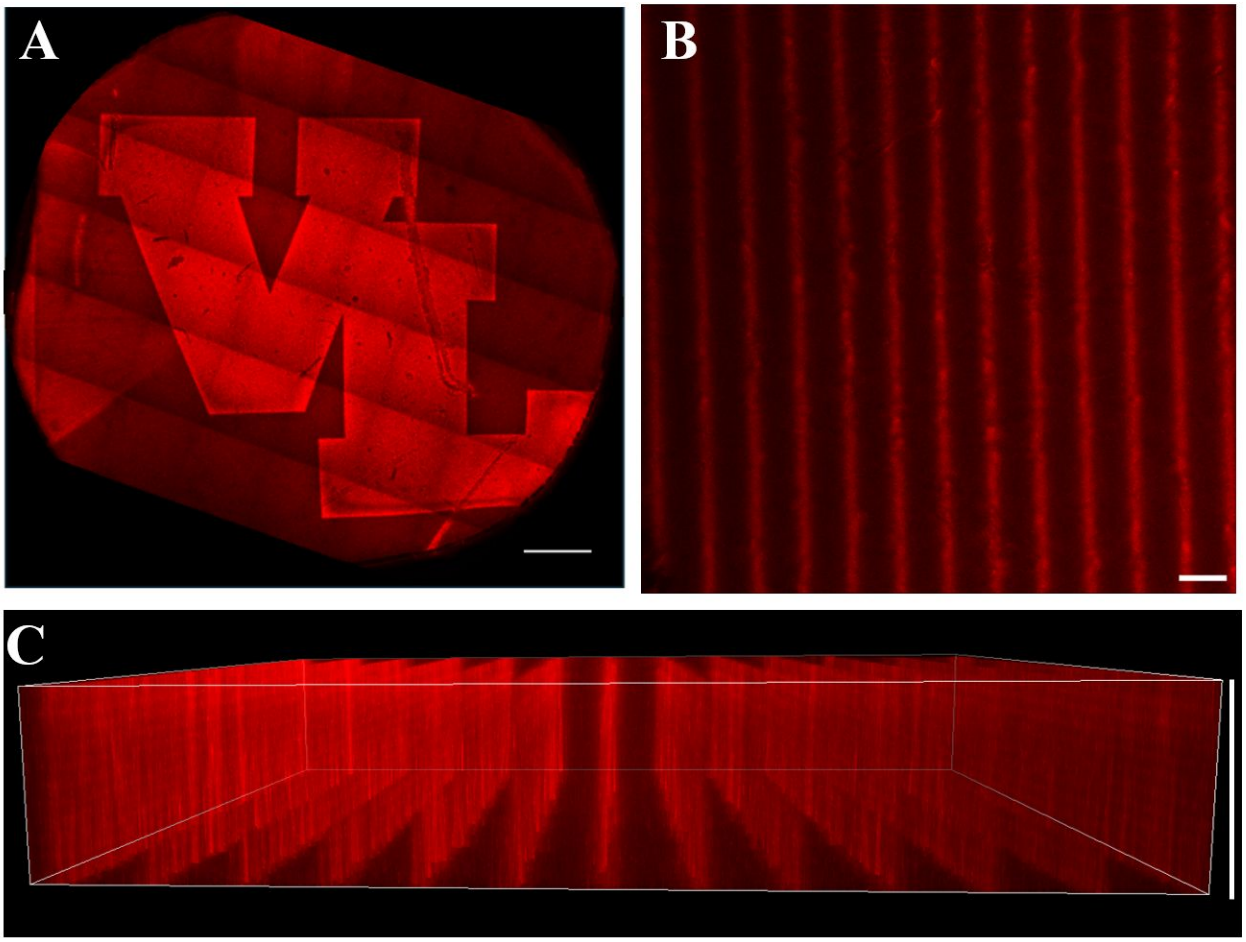

Supplemental Figure S15: Mono-Thiolated Rhodamine Peptide Patterning of HyaNor Hydrogels. (A) To demonstrate peptide photopatterning at the millimeter scale, a "VL" photomask (7.0 by 5.8 mm) was used to pattern the Vega Lab logo onto a cylindrical (8 mm diameter) HyaNor hydrogel. **(B)** To demonstrate peptide photopatterning at the micrometer scale, a striped photomask was used to pattern vertical stripes (10 µm width, 40 µm spacing) onto HyaNor hydrogels. **(C)** Thiolated rhodamine stripes penetrate at least 100 µm into HyaNor hydrogels, showing no visible depth-dependent differences in florescence. Scale bars: A = 1 mm, B = 50 µm, C = 100 µm.

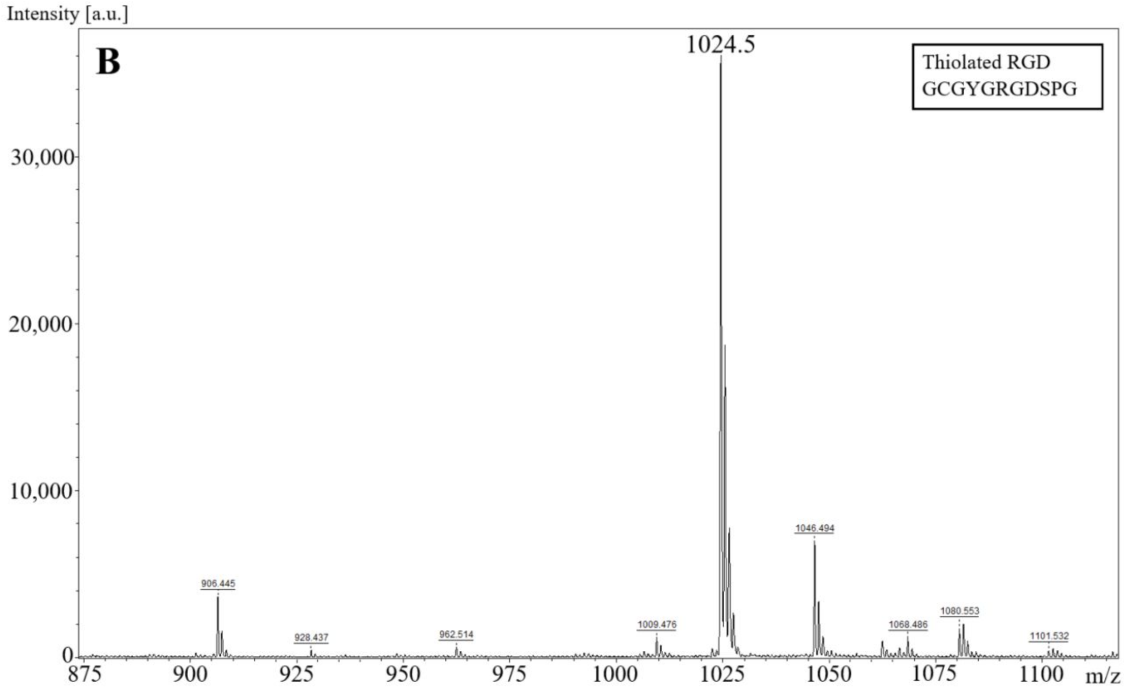

Supplemental Figure S16: MALDI-TOF Spectrometry of Thiolated RGD Peptides. Thiolated RGD peptide MALDI-TOF mass spectrometry results. Most intense peak: 1024.5 *m/z*; expected peak: 1024.4 *m/z*.

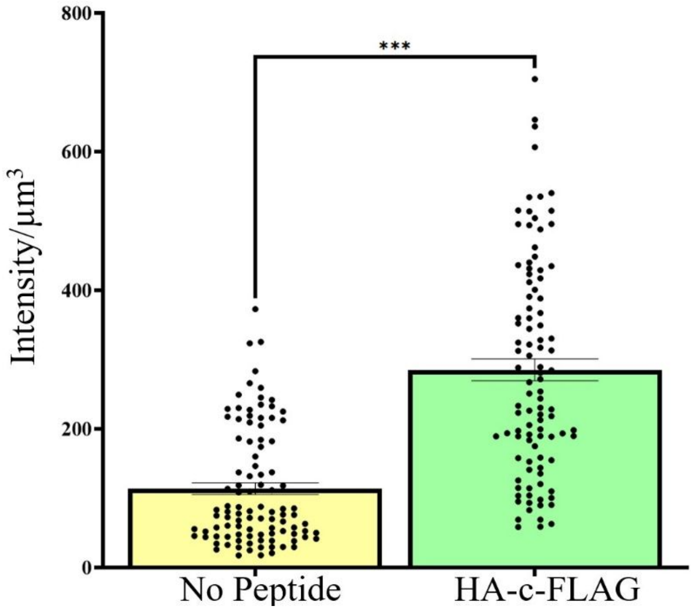

Supplemental Figure 17: 3D Image Analysis in Volumetric Units. Data from Figure of cells found within and outside the stimulatory HA-c-FLAG peptide region in terms of total green fluorescence intensity per µm^3 rather than in terms of voxels.

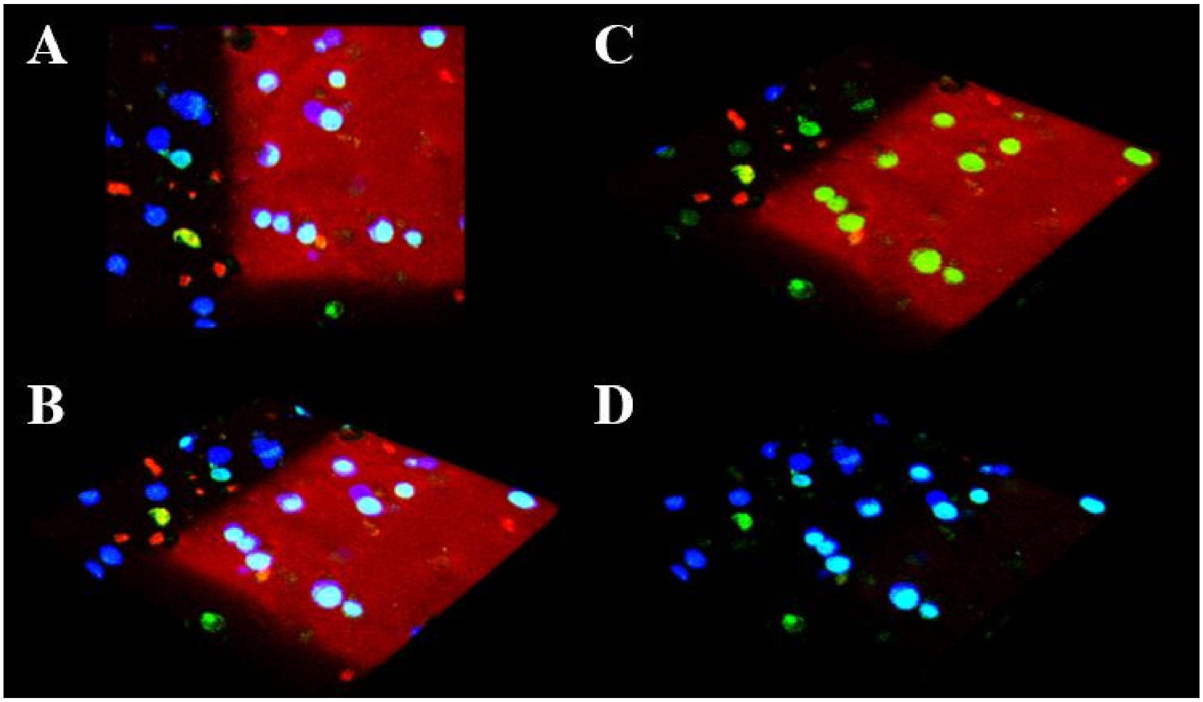

Supplemental Figure 18: 3D Visualization of Stimulatory EDPA Cells Spatially Responding to Tethered Stimulatory Peptides. (A) Top-down image of area featuring rhodamine squares (red), transfected cells (blue), and activated cells (green). **(B)** Angled view to show dimensionality. **(C)** Removal of blue channel to show green potency within and outside of stimulatory red square region. **(D)** Removal of red channel to visualize cell fluorescence.

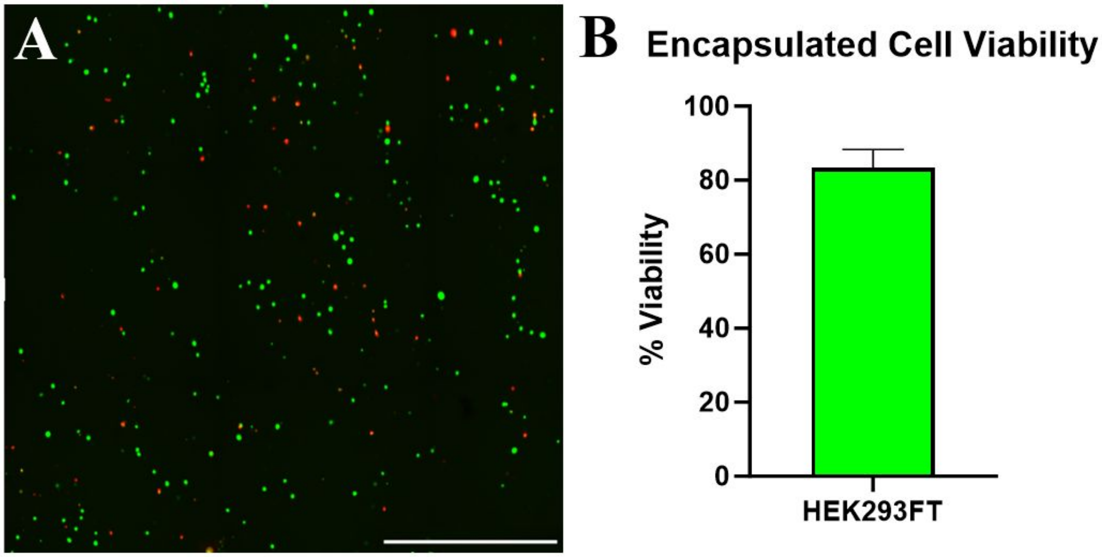

Supplemental Figure S19: Encapsulated Transfected Cell Viability. (A) Visualization of live (green) and dead (red) HEK293 transfected cells encapsulated in a 3D HyaNor hydrogel with 100 µM HA-c-FLAG peptide for 48 hours. Magnification 10x, scale bar: 1mm. **(B)** analysis of cell viability showing that 83% of cells were viable after this process. Error bar represents standard deviation. At least 100 cells were counted per hydrogel, n=3 hydrogels.

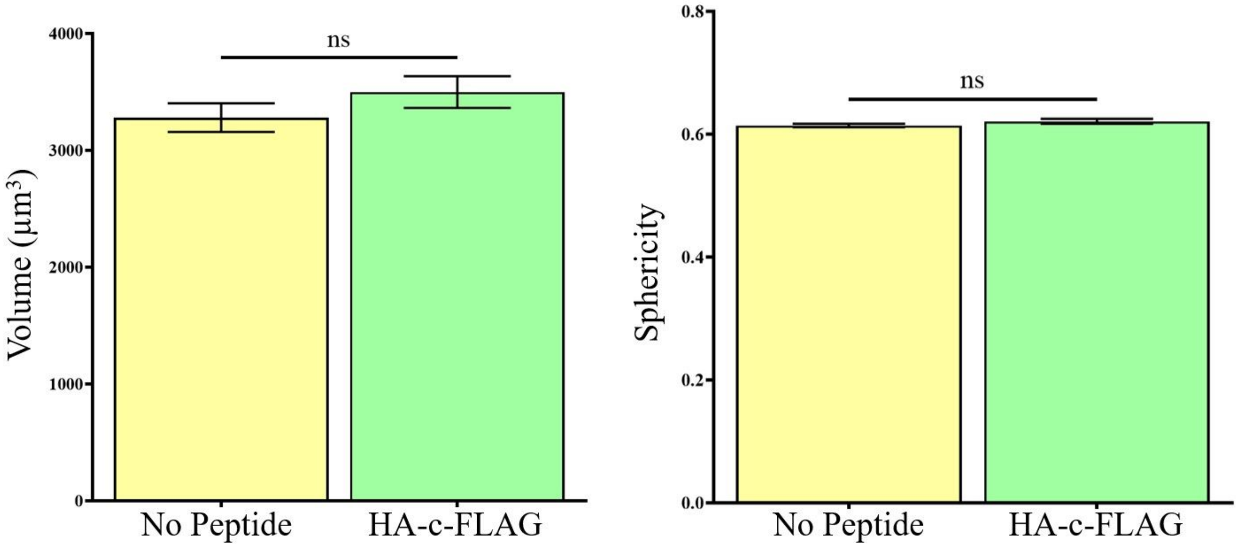

Supplemental Figure S20: 3D Morphology of Stimulatory EPDA-Programmed HEK293 Cells in the Absence or Presence of Tethered HA-c-FLAG Peptide Ligands. Cell morphology (volume, sphericity) of stimulatory EPDA-programmed HEK293 cells does not change in the absence (un-patterned area, red bar) or presence (peptide-patterned area, green bar) of tethered stimulatory peptide ligands within 3D HyaNor peptide-patterned hydrogels. Error bars represent standard deviation; ns denotes no statistical significance across groups. Error bars represent standard error around the mean (s.e.m.); ns $p > 0.05$. n=100 cells for each group.

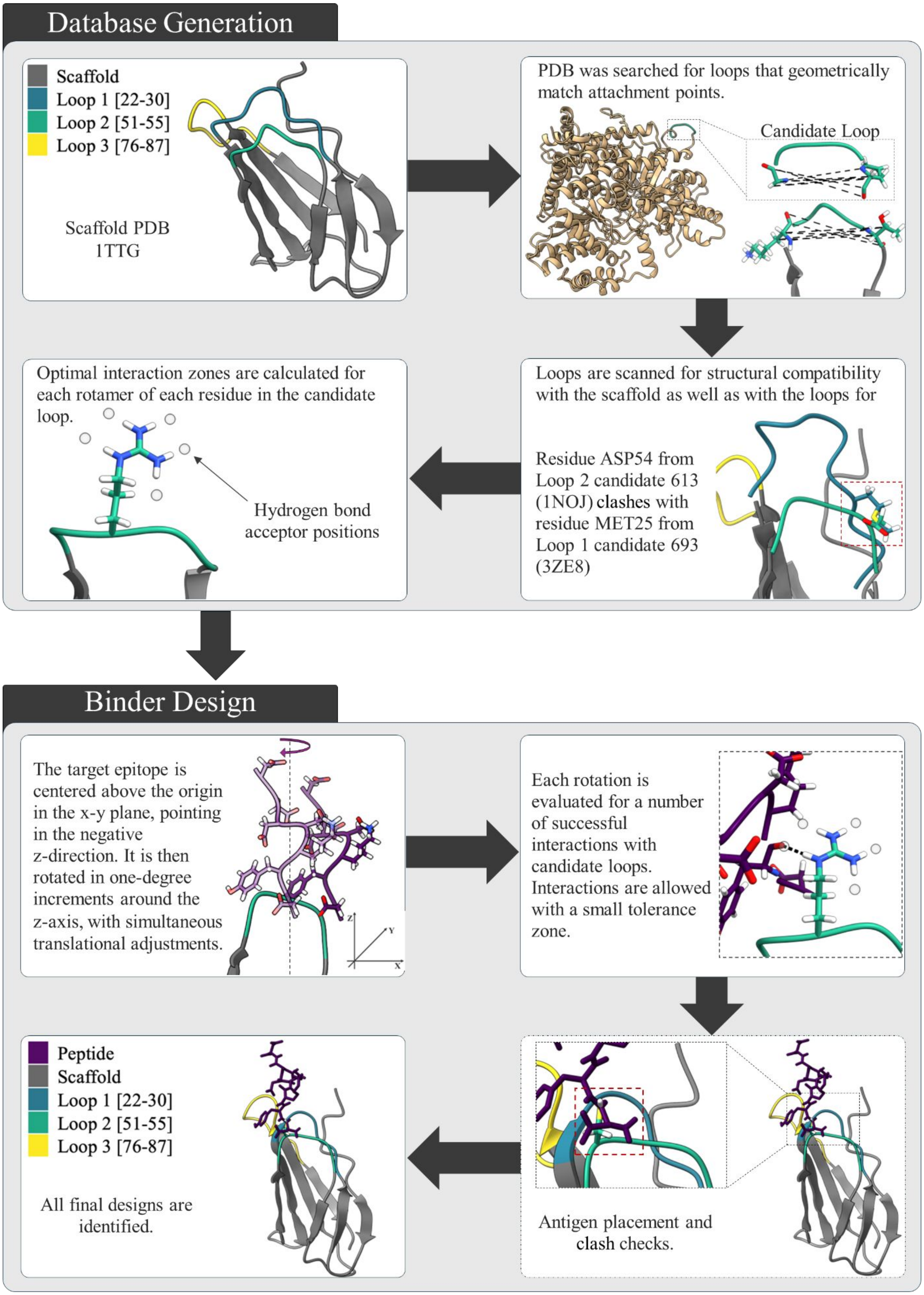

Database Generation
Scaffold
Loop 1 [22-30]
Loop 2 [51-55]
Loop 3 [76-87]
Scaffold PDB 1TTG
PDB was searched for loops that geometrically match attachment points.
Candidate Loop
Loops are scanned for structural compatibility with the scaffold as well as with the loops for
Residue ASP54 from Loop 2 candidate 613 (1NOJ) clashes with residue MET25 from Loop 1 candidate 693 (3ZE8)
Optimal interaction zones are calculated for each rotamer of each residue in the candidate loop.
Hydrogen bond acceptor positions
Binder Design
The target epitope is centered above the origin in the x-y plane, pointing in the negative z-direction. It is then rotated in one-degree increments around the z-axis, with simultaneous translational adjustments.
Each rotation is evaluated for a number of successful interactions with candidate loops. Interactions are allowed with a small tolerance zone.
Antigen placement and clash checks.
Peptide
Scaffold
Loop 1 [22-30]
Loop 2 [51-55]
Loop 3 [76-87]
All final designs are identified.

Supplemental Figure 21: Graphical Representation of the Database Generation and Binder Design Steps of PETEI. PETEI has two main steps, database generation and binder design. Database generation begins with the user identifying the scaffold protein for the designs and the loops in its structure that should be replaced. In this study, the scaffold protein was chain A from PDB file 1TTG and the loops were residues 22-30, 51-55, and 76-87. The loops connect to the scaffold at attachment point residues, which were residues 21, 31, 50, 56, 75, and 88 for this work. The scaffold is moved so that the centroid of the attachment point residues is at the origin and the loops point in the positive z-direction. Proteins from the PDB are then searched for loops that match the geometries of the attachment points. For example, residues 317 to 321 in chain A of PDB file 1NOJ were identified structure 613 / 708 for Loop 2. Note that structure 613 for Loop 2 is part of the FLAG5 solution. After candidate loops are identified, they are checked for clashes with structures for other loops. For example, Loop 2 structure 613 has a clash with Loop 1 structure 693, so those two loop structures are excluded from ever appearing in a solution together. Database generation concludes by calculating the spatial positions of all thermodynamically optimal interactions around the loop structures. PETEI does not allow mutations to positions, but it does use the Dunbrack Rotamer Library to allow for sampling alternative side chain positions. Shown are the positions of where hydrogen bond acceptors should be located around one of the rotamers of R53 of Loop 2 structure 613.

Binder design begins with the user specifying the protein to be bound and the specific residues that make up the target epitope. The target protein is moved so that the center of mass of the epitope is at 0.0, 0.0, 20.0 Å with the epitope residues pointing in the negative z-direction (i.e., towards the binding protein). The target protein is then rotated in 1 degree increments around the z-axis. For each orientation, the spatial positions of where optimal interactions can occur around the epitope are calculated. This is followed by identifying spatial translations of the target protein that will create at least a minimum number (e.g., 4) of exceptional interactions with binding protein loops. For example, in FLAG5 R53 of Loop 2 structure 613 forms a hydrogen bond with Y2 of the FLAG peptide. After a candidate solution is identified, it is checked for bad features, which include steric clashes, charge-charge clashes, and having too many positively charged residues in the designed binding loops. Designs that have enough excellent interactions and no detrimental features are considered successful designs without further evaluation (e.g., PETEI does not predict binding energies or do affinity maturation).

Table S1. Amino acid sequence and predicted binding metric value in kcal/ Å^2 for each of the HA, FLAG and MYC monobodies tested. From these, HA3, FLAG5, and MYC7 were determined to work best as extracellular receptor binders to their corresponding peptide ligand.

Receptor Name	Epitope Tag Peptide Recognition Sequence	Binding Metric Value (**w**)	Amino acid sequence
**HA 1**	**DYPYDVPDYA**	-0.04659	VSDVPRDLEVVAATPTSLLISNTVTGSYYRITYGET GGNSPVQEFTVGEKNPCLKETATISGLKPGVDYTIT VYAVNRNSPENLYISINYRT
**HA 2**	**DYPYDVPDYA**	-0.04632	VSDVPRDLEVVAATPTSLLISVSATKVKYYRITYGE TGGNSPVQEFTVESKTATATISGLKPGVDYTITVYA VTSVDHIFVAISINYRT
**HA 3**	**DYPYDVPDYA**	-0.04482	VSDVPRDLEVVAATPTSLLISINIGSKVNYYRITYGE TGGNSPVQEFTVRWGIATATISGLKPGVDYTITVYA VPKAAELANLINISINYRT
**HA 4**	**DYPYDVPDYA**	-0.04322	VSDVPRDLEVVAATPTSLLISVSATKVKYYRITYGE TGGNSPVQEFTVDLKLDTATISGLKPGVDYTITVYA VNQFNTNQLFVISINYRT
**HA 5**	**DYPYDVPDYA**	-0.04222	VSDVPRDLEVVAATPTSLLISIYGATQYYRITYGET GGNSPVQEFTVRWGIATATISGLKPGVDYTITVYAV GRRGAPVISINYRT
**HA 6**	**DYPYDVPDYA**	-0.04185	VSDVPRDLEVVAATPTSLLISADLAGAQYYRITYGE TGGNSPVQEFTVIDQTTTATISGLKPGVDYTITVYA VIRATNPPLLLAISINYRT
**HA 7**	**DYPYDVPDYA**	-0.04174	VSDVPRDLEVVAATPTSLLISAAVGPFTYYYRITYG ETGGNSPVQEFTVRNNRITATISGLKPGVDYTITVY AVIKGDDELFVGISINYRT
**HA 8**	**DYPYDVPDYA**	-0.04095	VSDVPRDLEVVAATPTSLLISITAPAGTTYYRITYGE TGGNSPVQEFTVQKGVTTATISGLKPGVDYTITVYA VGKYWDAGISINYRT
**HA 9**	**DYPYDVPDYA**	-0.04092	VSDVPRDLEVVAATPTSLLISCYGFSRYYRITYGET GGNSPVQEFTVRHGIPTATISGLKPGVDYTITVYAV QWRHSYTAISINYRT
**HA 10**	**DYPYDVPDYA**	-0.04067	VSDVPRDLEVVAATPTSLLISNTVTGSYYRITYGET GGNSPVQEFTVQKGVTTATISGLKPGVDYTITVYA VSINNETPGIRISINYRT
**FLAG 1**	**DYKDDDDK**	-0.05695	VSDVPRDLEVVAATPTSLLISSARGGSYYYRITYGE TGGNSPVQEFTVLLRVPTATISGLKPGVDYTITVYA VIKDGVMVLISINYRT
**FLAG 2**	**DYKDDDDK**	-0.05640	VSDVPRDLEVVAATPTSLLISLGIYGKVNIHYYRITY GETGGNSPVQEFTVKIGMPTATISGLKPGVDYTITV YAVLKSGFLQISINYRT
**FLAG 3**	**DYKDDDDK**	-0.05450	VSDVPRDLEVVAATPTSLLISIVIRAKYYRITYGETG GNSPVQEFTVSFDYSTATISGLKPGVDYTITVYAVA KEGVTWSISINYRT
**FLAG 4**	**DYKDDDDK**	-0.05430	VSDVPRDLEVVAATPTSLLISIVTNGAYYRITYGET GGNSPVQEFTVEYRNTQTATISGLKPGVDYTITVYA VGKYHPHGDISINYRT
**FLAG 5**	**DYKDDDDK**	-0.05385	VSDVPRDLEVVAATPTSLLISVSATKVKYYRITYGE TGGNSPVQEFTVGCRDPTATISGLKPGVDYTITVYA VVQNGEYDLNAISINYRT

**FLAG 6**	**DYKDDDDK**	-0.05385	VSDVPRDLEVVAATPTSLLISVSATKVKYYRITYGETGGNSPVQEFTVGKISCTATISGLKPGVDYTITVYAVLYQDITLSISINYRT
**FLAG 7**	**DYKDDDDK**	-0.05211	VSDVPRDLEVVAATPTSLLISCQYTGNPIKYYRITYGETGGNSPVQEFTVRHGIPTATISGLKPGVDYTITVYAVLGRNDAIGISINYRT
**FLAG 8**	**DYKDDDDK**	-0.05208	VSDVPRDLEVVAATPTSLLISSVFVACRYYRITYGETGGNSPVQEFTVRWGIATATISGLKPGVDYTITVYAVPSGGGDPVISINYRT
**FLAG 9**	**DYKDDDDK**	-0.05200	VSDVPRDLEVVAATPTSLLISSHTGGHASYYRITYGETGGNSPVQEFTVNKRQLTATISGLKPGVDYTITVYAVGNGKFNLMISINYRT
**FLAG 10**	**DYKDDDDK**	-0.05166	VSDVPRDLEVVAATPTSLLISHSAAFEDLRYYRITYGETGGNSPVQEFTVKTGQPTATISGLKPGVDYTITVYAVDHHDIVGWISINYRT
**MYC 1**	**EQKLISEEDL**	-0.04191	VSDVPRDLEVVAATPTSLLISCNLSLISYYRITYGETGGNSPVQEFTVEYRNTQTATISGLKPGVDYTITVYAVREDGSFLIISINYRT
**MYC 2**	**EQKLISEEDL**	-0.03856	VSDVPRDLEVVAATPTSLLISASIGANSTYYRITYGETGGNSPVQEFTVGARGYETATISGLKPGVDYTITVYAVLKGKNQAFIISINYRT
**MYC 3**	**EQKLISEEDL**	-0.03498	VSDVPRDLEVVAATPTSLLISVSATKVKYYRITYGETGGNSPVQEFTVSASTATATISGLKPGVDYTITVYAVAKEGVTWSISINYRT
**MYC 4**	**EQKLISEEDL**	-0.03483	VSDVPRDLEVVAATPTSLLISVSATKVKYYRITYGETGGNSPVQEFTVEYHNTQTATISGLKPGVDYTITVYAVPKAAELANLINISINYRT
**MYC 5**	**EQKLISEEDL**	-0.03464	VSDVPRDLEVVAATPTSLLISIVLVNPKYYRITYGETGGNSPVQEFTVGGNNPTATISGLKPGVDYTITVYAVPLLGIPVAISINYRT
**MYC 6**	**EQKLISEEDL**	-0.03455	VSDVPRDLEVVAATPTSLLISGKVETNEYYRITYGETGGNSPVQEFTVNKRQLTATISGLKPGVDYTITVYAVANIPGCNPLISINYRT
**MYC 7**	**EQKLISEEDL**	-0.03446	VSDVPRDLEVVAATPTSLLISVSAINVKYYRITYGETGGNSPVQEFTVLLRVPTATISGLKPGVDYTITVYAVLNDTYSVYISINYRT
**MYC 8**	**EQKLISEEDL**	-0.03431	VSDVPRDLEVVAATPTSLLISVSATKVMYYRITYGETGGNSPVQEFTVTFRISTATISGLKPGVDYTITVYAVGNGKFNLMISINYRT
**MYC 9**	**EQKLISEEDL**	-0.03416	VSDVPRDLEVVAATPTSLLISTDFHNVKYYRITYGETGGNSPVQEFTVVATNTATISGLKPGVDYTITVYAVGNGKFNLMISINYRT
**MYC 10**	**EQKLISEEDL**	-0.03413	VSDVPRDLEVVAATPTSLLISLNKCDMVDYYRITYGETGGNSPVQEFTVPNGTRTATISGLKPGVDYTITVYAVGSSSRPNSGLAISINYRT
